# Electrochemical synthesis of copper(i) acetylides *via* simultaneous copper ion and catalytic base electrogeneration for use in click chemistry†

**DOI:** 10.1039/c9ra06782e

**Published:** 2019-09-17

**Authors:** Peter W. Seavill, Katherine B. Holt, Jonathan D. Wilden

**Affiliations:** Department of Chemistry, University College London 20 Gordon Street London WC1H 0AJ UK pseavill@gmail.com j.wilden@ucl.ac.uk

## Abstract

We report an efficient and sustainable electrochemical synthesis of copper(i) acetylides using simultaneous copper oxidation and Hofmann elimination of quaternary ammonium salts. The electrochemically-generated base was also regenerated electrochemically, making it catalytic. A ‘Click test’ (CuAAC reaction) was performed to assess product purity and an electrochemically-promoted, one-pot CuAAC reaction was performed, which serves as a promising initial demonstration of this approach in a pharmaceutically-relevant reaction.

## Abstract 2

We report an efficient and sustainable electrochemical synthesis of copper(i) acetylides using simultaneous copper oxidation and Hofmann elimination of quaternary ammonium salts.

## Graphical abstract

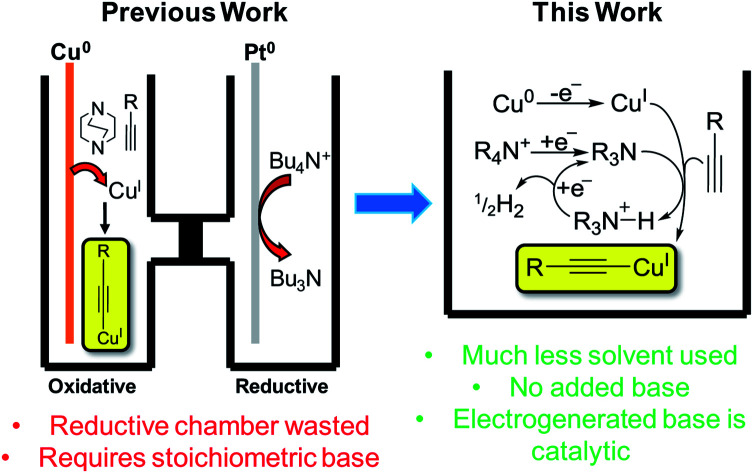

## Introduction

**Fig. 1:**
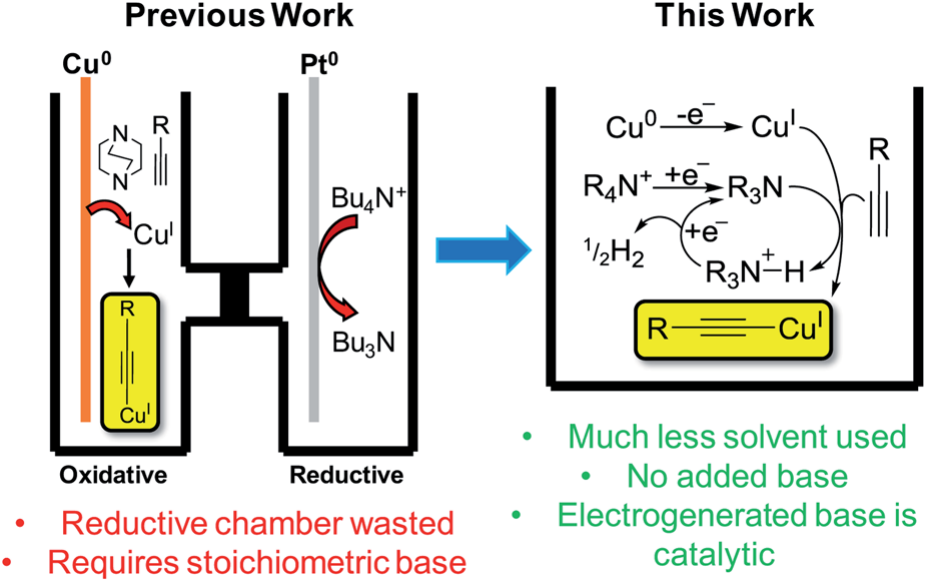
Previous electrochemical copper(i) acetylide synthesis and proposed improvements.^2^

Copper has great potential utility in electro-organic chemistry due to its readily accessible redox states. The application of mild electrical potentials to exert control over oxidation states of copper catalysts introduced to solutions has previously been exploited to select for either Glaser–Hay (Cu^II^ pathway) or CuAAC (Cu^I^ pathway) reactions.^1^ However, the use of elemental copper as an electrode material to produce Cu^I^ ions *in situ* for reactions has only recently been published by our group.^2^ This work represented an electrochemical synthesis and isolation of copper(i) acetylides, which are valuable intermediates in many synthetic processes, such as Huisgen-type/Click,^3^ Castro–Stephens,^4^ halogenation,^3^ Sonogashira,^5^ ynamide-formation^6^ and phosphorus-substitution reactions,^6^ as well as for the formation of a variety of products *via* photochemical protocols.^7^ Traditionally prepared by reacting a terminal alkyne with a copper halide in aqueous ammonia with EtOH or in DMF with K_2_CO_3_,^8^ we found that in a divided cell, applying a positive potential whilst using a Cu^0^ working-electrode, having DABCO present as a base and using Bu_4_NPF_6_/MeCN as an electrolyte solution, we could efficiently produce Cu^I^ ions that were used to form the desired copper(i) acetylides in excellent yields. An electrochemical synthesis has advantages over traditional methods in terms of sustainability, particularly in removing halide waste from the process entirely.^2^ In this current work, we aimed to develop this process further. We hypothesised that we could carry out this reaction in an undivided cell by incorporating the reduction reaction of the tetrabutylammonium (TBA) electrolyte salt used in our previous conditions to produce Bu_3_N *in situ* and obviate the requirement for any added base, such as DABCO, in our protocol. Furthermore, we hoped to demonstrate and make use of a catalytic base cycle by electrochemically reducing protonated base species, releasing only H_2_ gas as a clean by-product,^9^ improving the efficiency of this method further still (Fig. 1).

**Scheme 1:**
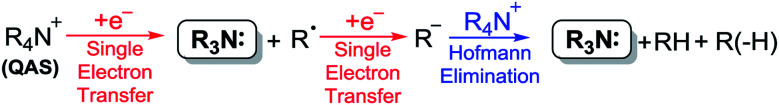
Quaternary ammonium salt (QAS) reduction.^11^

It is well-understood in the literature that the generation of various carbon, oxygen and nitrogen-centred anions and radical anions *via* cathodic reduction of appropriate probases can be used to promote reactions in a basic fashion.^10^ Many of these reductions are carried out in the presence of quaternary ammonium salts (QAS), in particular, tetraethylammonium (TEA) and TBA salts, which are commonly used in electrochemical cells as background electrolytes. Importantly, in the absence of common probases, QAS can themselves be reduced by single-electron-transfer/Hofmann-type elimination processes to generate tertiary amine bases (Scheme 1).^11^

By comparison to most probases, QAS are more resistant to electrochemical reduction; the effects of chain length, branching and steric hindrance having very little effect on both their stability towards reduction and the electronic environment around their cationic nitrogen centres.^12^ Such features are generally desirable for their use as ‘inert’ electrolytes. However, there are benefits to employing such salts as both the background electrolytes and as probases in electrochemical systems, namely, the increased sustainability incurred from omitting any additional base or probase reagents. The advantages to using an electrochemical approach to generate bases *in situ* over non-electrochemical methods are that many QAS are less hazardous than their tertiary amine counterparts, through careful selection of anions used, making the associated risks of the starting materials preferable. There is also the potential to use bases catalytically by electro-regeneration of the base species.^9^ Such factors embody several of the key principles of green chemistry,^13^ yet whilst examples of electro-reduction of QAS exist,^14^ to the best of our knowledge, none have been utilised specifically for the *in situ* production of tertiary amine bases.

## Results and discussion

We began by using similar reagents to our previous method,^2^ therefore Bu_4_NPF_6_/MeCN was used as the electrolyte solution, causing Cu^I^ to be produced from the Cu^0^ working electrode (WE) and (so we initially believed) Bu_3_N to be formed directly at the Pt counter electrode (CE). Over the course of 2 h of applied potential (+0.50 V *vs.* Ag wire quasi-reference electrode (QRE)), a modest yield of 54% for 1a was achieved (Table 1). The reaction vessel was kept under argon to prevent any diyne 2 forming *via* the Cu^II^-promoted Glaser–Hay reaction.^15^ To demonstrate the proposed catalytic nature of the base, 0.1 mmol electrolyte was used with respect to 0.3 mmol phenylacetylene, hence, if all QAS was converted into tertiary amine bases 3 or 4, a maximum theoretical yield for 1a of 33% is predicted. Yields greater than this demonstrate the base must be electrochemically regenerated after initial deprotonation of a molecule of alkyne (Fig. 1).

**Table 1:** Optimisation and control reactions^a^

Entry	Electrolyte/solvent used	Voltage (*vs.* Ag QRE) and total charge passed	Additive(s)	Yield^b^/%
1	Bu_4_NPF_6_/MeCN	+0.50 V for 2 h, 19.2C passed	—	54
2	Bu_4_NPF_6_/MeCN	No potential applied (20 h)	—	0
3	MeCN	No potential applied (2 h)	Cu(MeCN)_4_PF_6_ (1.1 eq.), Bu_3_N (0.33 eq.)	3
4	MeCN	No potential applied (2 h)	Cu(MeCN)_4_PF_6_ (1.1 eq.), Bu_3_N (1.1 eq.)	38
5	LiClO_4_/MeCN	+0.50 V for 2 h, 14.8C passed	—	0
6	LiClO_4_/MeCN	+0.50 V for 2 h, 5.0C passed	Bu_3_N (0.33 eq.)	9
7	Et_4_N(CH_3_C_6_H_4_SO_3_)/MeCN	+0.50 V for 2 h, 19.0C passed	—	66
8	Et_4_N(CH_3_C_6_H_4_SO_3_)/MeCN	No potential applied (2 h)	—	<1
9	MeCN	No potential applied (2 h)	Cu(MeCN)_4_PF_6_ (1.1 eq.), Et_3_N (0.50 eq.)	44
10	MeCN	No potential applied (2 h)	Cu(MeCN)_4_PF_6_ (1.1 eq.), Et_3_N (1.1 eq.)	51
**11**	**Et**_**4**_ **N(CH**_**3**_ **C**_**6**_ **H**_**4**_ **SO**_**3**_ **)/MeCN**	**+0.50 V for 4 h, 45.7C passed**	**—**	**97**
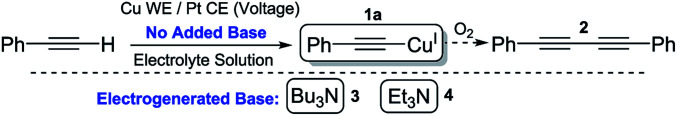				

a In all cases 0.3 mmol phenylacetylene and 0.1 mmol electrolyte salt in 10 mL reagent grade MeCN (0.01 M) were used. All reactions carried out under argon with a Cu wire WE, a Pt wire CE and a Ag wire QRE each with an effective surface area of 64 mm^2^.

b Isolated yield of copper acetylide 1a.

We propose that the active Cu species in this reaction is Cu(MeCN)_4_X (where X = PF_6_^−^ or CH_3_C_6_H_4_SO_3_^−^) based on our previous work and supported again by control reactions carried out in this work. Entry 3 shows that without an applied potential, the reaction proceeded when this Cu species was added along with an amount of 3 that mirrored the total available QAS used in entry 1 (*i.e.* 0.33 eq. with respect to the alkyne), although the reaction was much less efficient. Furthermore, it was found that when a stoichiometric/slight excess of 3 was used the yield increased dramatically. This further indicates that when a potential is applied, the base is regenerated, making this process catalytic in nature.

**Scheme 2:**
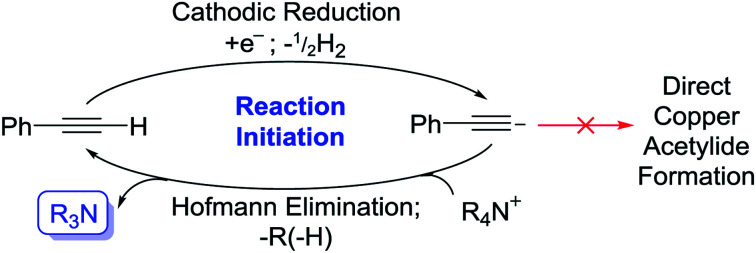
Proposed reaction initiation.

The absence of any appropriate QAS probase (LiClO_4_ used as substitute) completely shut the reaction down even when a potential was applied (1a was not produced over the 2 h electrolysis) as shown in entry 5. However, when Bu_4_NPF_6_ was added to this same solution and a potential (+0.5 V *vs.* Ag QRE) was applied again, within 15 min a bright yellow precipitate of 1a was produced. Whilst we initially interpreted this to be evidence of direct electrochemical reduction of a QAS as in Scheme 1, we decided to run cyclic voltammetry (CV) plots of the various components of this reaction mixture to obtain evidence for this hypothesis (CV plots shown in the ESI and Fig. S2–S4†). Fig. S2† appears to show that at around −3.0 V (*vs.* Ag QRE) the background electrolyte solution begins to be reduced. It has been reported that under a reducing potential MeCN itself can form a strong base, [NCCH_2_]^−^,^16^ which has been shown to be capable of initiating β-lactam synthesis through substrate deprotonation.^16*b*–*d*^ However, this direct reduction of MeCN appears to only take place in the absence of other proton donors,^16*a*^ suggesting that the reduction peak shown in these CV plots likely relates to QAS reduction. This distinction is rendered somewhat moot by the fact that at the lower potential of −2.2 V, phenylacetylene starts to be reduced to [PhCC]^−^ (Fig. S3†), showing that under the conditions used here, this is the most likely first reductive process to take place. Deprotonation of QAS *via* Hofmann elimination would then produce a stable tertiary amine base, thereby initiating the copper acetylide-producing reaction. The subsequent electrochemical reduction of any protonated tertiary amine bases would then almost certainly take over as the dominant reductive process for the rest of the reaction. It is not immediately apparent as to why the production of the phenylacetylide anion does not directly lead to the formation of 1a. One explanation could be that this reactive anion (formed in low concentration at the beginning of the electrolysis) is quenched too quickly to react directly with the similarly low concentration of Cu^I^ ions produced. The stable bases 3 and 4, produced by way of Hofmann elimination, would not suffer from this problem. Scheme 2 shows this proposed reaction initiation.

Entries 5 and 6 also proved important for other reasons. Given that these reactions were carried out in the presence of reagent grade (rather than anhydrous) MeCN, it was postulated that a build-up of hydroxide ions was possible. This could facilitate the reaction by providing another base for the deprotonation step of the reaction and increase the rate at which 3 or 4 were regenerated by deprotonating any protonated 3 or 4. Entries 5 and 6 seem to suggest that these processes were not in effect.

**Scheme 3:**
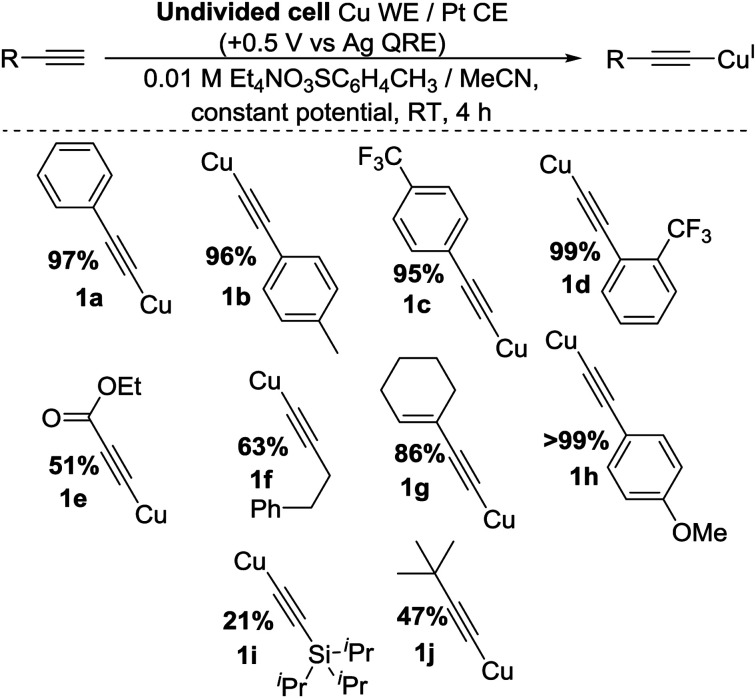
General conditions and scope of reaction.

To improve the yield and atom efficiency of the reaction, we tested an alternative electrolyte salt, Et_4_N(O_3_SC_6_H_4_CH_3_), aiming to produce the less sterically-hindered base 4. Work carried out by Dahm and Peters^11*a*^ makes it clear that during the formation of 3 from TBA^+^, a sterically-demanding gauche interaction must exist in order to obtain the necessary antiperiplanar geometry required in Hofmann elimination processes. However, this same interaction is much smaller when using TEA^+^, promoting the generation of 4 much more readily than 3. Confirming this hypothesis, the move over to this TEA salt increased the yield significantly, the catalytic nature of the base was maintained and as this salt was more atom-efficient we continued its use. We also found that for optimal yields of 1a the potential should be applied for 4 h, giving us our optimised conditions as shown in entry 11 of Table 1, highlighted in yellow. Applying these conditions to a range of substrates proved successful, as shown in Scheme 3, with yields comparing well with classical literature methods and a variety of substituents and functional groups being tolerated. However, we found that when trimethylsilyl acetylene was used, the product appeared to decompose *in situ*, presumably due to exposure to the reducing counter electrode. This contrasts our previous method.^2^ A bulkier silane, 1i, was produced, albeit in low yield.

Initially we found that some substrates gave impure products when reagent grade MeCN was employed, likely due to overoxidation of the copper. To remedy this, we switched to anhydrous MeCN and obtained superior results.

**Fig. 2:**
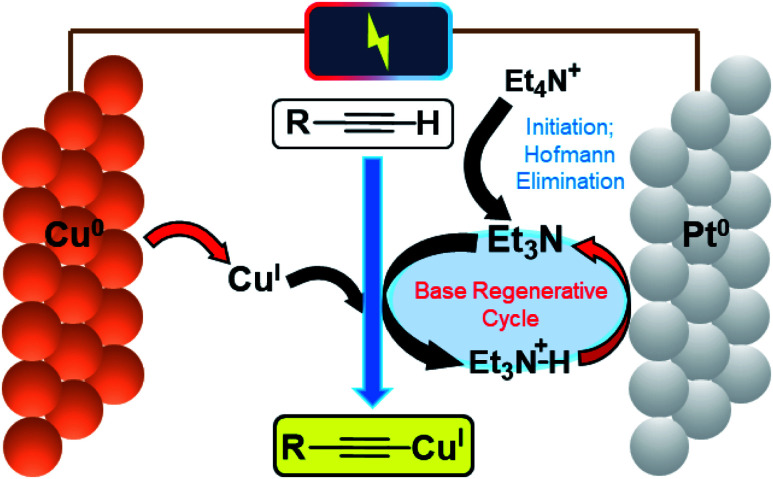
Schematic mechanism of electrochemical Cu^I^ and base generation/catalytic regeneration.

A schematic mechanism for this reaction is given in Fig. 2, highlighting the various single-electron-transfer redox reactions taking place at electrode surfaces (red arrows).

**Fig. 3:**
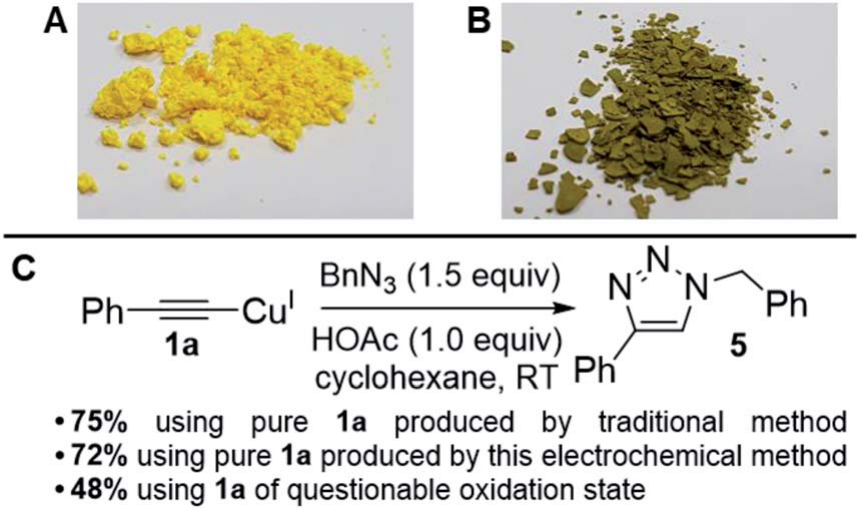
(A) Picture of 1a that matches literature physical descriptions. (B) Picture of 1a that is of questionable oxidation state. (C) ‘Click test’ of copper acetylides to assess product purity.^19^

The generally high yields, absence of any detectable diyne by-products and lack of degradation of the materials post reaction suggested that our products were indeed pure, but to rigorously test our copper acetylides and the validity of our method, we performed a simple Huisgen-type reaction (the most famous of the ‘Click’ reactions)^17^ to form a 1,2,3-triazole product *via* Cu-promoted azide–alkyne coupling (CuAAC) which proceeds through a copper(i) acetylide intermediate. This reaction is a good exemplar because it is so widely-used, especially in pharmaceutical chemistry where many drug molecules, biomaterials and polymers are routinely produced using this chemistry.^18^ It is also a reaction known to be efficient and relies upon a Cu^I^-based catalytic cycle, meaning that if our copper acetylides were in a mixed oxidation state, this would be highlighted clearly. We therefore adapted conditions from Shao *et al.*,^19^ deliberately selecting a method without a reducing agent such as sodium ascorbate to remove the possibility of Cu^II^ being converted into Cu^I^ mid-reaction (Fig. 3).

**Scheme 4:**
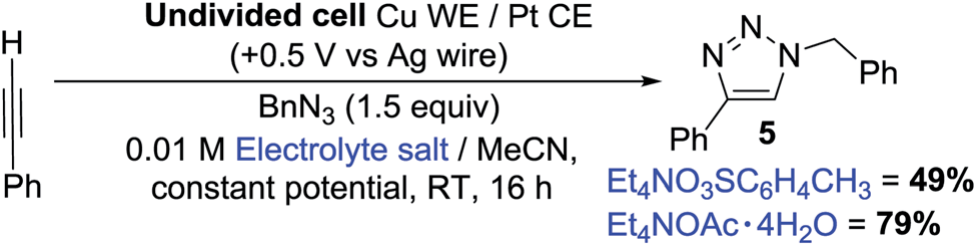
One-pot electrochemical CuAAC reaction.

To our delight, we found that the yields and spectral data for 5 produced using 1a from both the traditional method (synthesised using CuI in NH_3_–H_2_O–EtOH^8^) and our new electrochemical method matched very well, reaffirming that our new method for producing copper acetylides is robust. We also noted that when 1a of questionable oxidation state, *i.e.* a possible mixture of Cu^I^ and Cu^II^ acetylides as in picture B of Fig. 3 was used, a significantly lower yield of 48% was obtained for 5. Emboldened by these results, we attempted to integrate our electrochemical copper(i) acetylide formation with the Click reaction to produce a sustainable, one-pot electrochemical process, as shown in Scheme 4. Previously, groups have carried out electro-assisted CuAAC-type reactions on electrode surfaces coated with either alkyne or azide functional groups, where Cu(ii) salts added to solution are electrochemically reduced to Cu(i), initiating the Click reaction.^20^ Another approach involving the generation of the alkyne moiety on the surface of electrodes through the reduction of Co_2_(CO)_6_ has also been demonstrated,^21^ but to our knowledge this is the first example of both an electro-oxidised Cu(0) to Cu(i) approach and of such a reaction on preparative-scale. We obtained yields of 49% for 5 with Et_4_NO_3_SC_6_H_4_CH_3_ and 79% with Et_4_NOAc·4H_2_O (control reactions with no potential applied yielded 2% and 0% respectively). These results suggest that the presence of acetate anions permits the generation of a potent copper acetate catalyst, indeed, control reactions using Cu(i)OAc and Et_3_N produced 5, but in lower yields than the electrochemical method. Furthermore, trace amounts of diyne 2 were also produced (presumably from Cu(ii) contamination of the Cu(i)OAc catalyst) which was not observed in any of the electrochemical tests where Cu(i) is generated *in situ*.

## Conclusions

In summary, we have successfully improved the efficiency and sustainability of copper(i) acetylide synthesis using electrochemistry in an undivided cell. This decreased the amount of solvent required, the base was generated from the background electrolyte and regenerated electrochemically to make it catalytic and halogen waste was completely eliminated from the process. We rigorously assessed the fidelity of our products through a ‘Click test’ (CuAAC reaction) and we successfully integrated the two reactions into a sustainable, one-pot electrochemical process, which serves as a promising initial demonstration of this approach in a pharmaceutically-relevant reaction.

## Conflicts of interest

There are no conflicts to declare.

## Supplementary Material

RA-009-C9RA06782E-s001
