# Supplementary material for: Electrochemical synthesis of copper(i) acetylides via simultaneous copper ion and catalytic base electrogeneration for use in click chemistry
Source: RSC Adv. 2019 Sep 17;9(50):29300–4. doi: 10.1039/c9ra06782e (PMC9071997; doi:10.1039/c9ra06782e)
Supplement: RA-009-C9RA06782E-s001 [file RA-009-C9RA06782E-s001.pdf]

Electronic Supplementary Material (ESI) for RSC Advances.
This journal is © The Royal Society of Chemistry 2019

Electronic Supplementary Information for RSC Advances

Electrochemical Synthesis of Copper(I) Acetylides Via Simultaneous Copper Ion And Catalytic Base Electrogeneration For Use In Click Chemistry

Peter W. Seavill, Katherine B. Holt and Jonathan D. Wilden**

Department of Chemistry, University College London, 20 Gordon Street, London, WC1H 0AJ, UK.
*Corresponding authors, E-mail: pseavill@gmail.com, j.wilden@ucl.ac.uk

Table of Contents:

General Information: **3**

Details of Electrochemical Methods: **3**

Electrochemical Preparation of Copper Acetylides: **4**

Cyclic Voltammetry (CV) Plots of Phenylacetylene: **4**

General Electrochemical Procedure for Copper(I) Acetylide Synthesis: **6**

IR Spectra for Copper Acetylides: **8**

'Click test' of Copper(I) Acetylides Produced By Traditional Methods and Using the Electrochemical Method Developed in this Work: **13**

Traditional Procedure for Copper(I) Acetylide 1a Formation: (adapted from Evano *et al.*[7]) **13**

General Procedure for 1,2,3-Triazole Formation: (adapted from Shao *et al.*[8]) **13**

Electrochemical Procedure for One-Pot Click Reaction: **18**

References: **18**

General Information:

Solvents and reagents were purchased from suppliers and used without any further purification unless otherwise stated. Normal phase silica gel (Merck KGaA) and sand (VWR) were used for column chromatography. Reactions were monitored by TLC unless otherwise stated. TLC plates pre-coated with silica gel 60 F_{254} on aluminium (Merck KGaA) were used, detection by UV (254 nm) and chemical stain (potassium permanganate). Mass spectra were measured on Thermo Finnigan MAT900 XE and Waters LCT Premier XE machines operating in ESI mode. ^{1}H NMR spectra were recorded at 600 MHz and ^{13}C NMR spectra were recorded at 100 MHz on Bruker Avance spectrometers at ambient temperature. All chemical shifts were referenced to the residual proton impurity of the deuterated solvent. In ^{1}H NMR the multiplicity of the signal is indicated as s (singlet), d (doublet), t (triplet), q (quartet) and m (multiplet), defined as all multipeak signals where overlap or complex coupling of signals makes definitive descriptions of peaks difficult. The ^{13}C NMR is assigned as C (quaternary), CH, CH_2 and CH_3 as determined by DEPT 135. Coupling constants are defined as *J* and quoted in Hz to one decimal place. Infrared spectra were obtained on a Bruker Alpha FTIR Spectrometer operating in ATR mode and peaks were assigned as ‘w’, ‘m’ or ‘s’ denoting weak, medium or strong peaks respectively. Melting points were measured with a Gallenkamp apparatus and are uncorrected. Decomposition points were recorded on this same apparatus but should not be taken as definitive characterisation values due to their inherently large margins of error. *In vacuo* is used to describe solvent removal by Büchi rotary evaporation between 17-40 °C. For NMR experiments, $CDCl_3$ denotes deuterated (d_1) chloroform. Electrochemical reactions were carried out using an Ivium Technologies Vertex model potentiostat operating in chronoamperometry mode. CV plots were carried out using this same potentiostat with a glassy carbon working-electrode, a Pt wire counter-electrode and a Ag wire quasi reference-electrode.

Details of Electrochemical Methods:

The general experimental setup we used for electrochemical reactions was designed to be as simple and accessible as possible. In doing so we hope to minimise the disparity and lack of reproducibility of results inherent in electro-organic synthesis due to there being a lack of standardised experimental setups. For all reactions we used a 10 mL sample vial as our reaction vessel/undivided cell (dimensions shown in **Figure S1**) as a size B19 Suba-Seal fit perfectly in the neck to create a seal. A balloon with argon was fitted into the centre of the Suba-Seal for degassing and maintaining an anaerobic environment during electrolysis. Three metal wire electrodes were also inserted into the Sub-Seal in a triangular pattern, to a depth of 20 mm into the 10 mL electrolyte solutions used in the reactions. The electrodes were held at 7-10 mm distance between any two electrodes over the course of reactions. As the three electrodes used were the same diameter (1 mm), and all held at the same depth into the solution, they all had the same effective surface area of 64 mm^2. A copper wire was used as the working electrode (WE), a silver wire was used as a quasi reference-electrode (QRE) and a platinum wire was used as the counter-electrode (CE). Images of the assembled reaction vessel and a schematic view of the Suba-Seal are shown in **Figure S1**. Reactions were run using an Ivium Technologies Vertex model potentiostat operating in chronoamperometry mode. This model allowed for real-time charge over time and current over time graphs to be generated which we found exceedingly useful for this work, especially for measuring charge passed over the course of reactions.

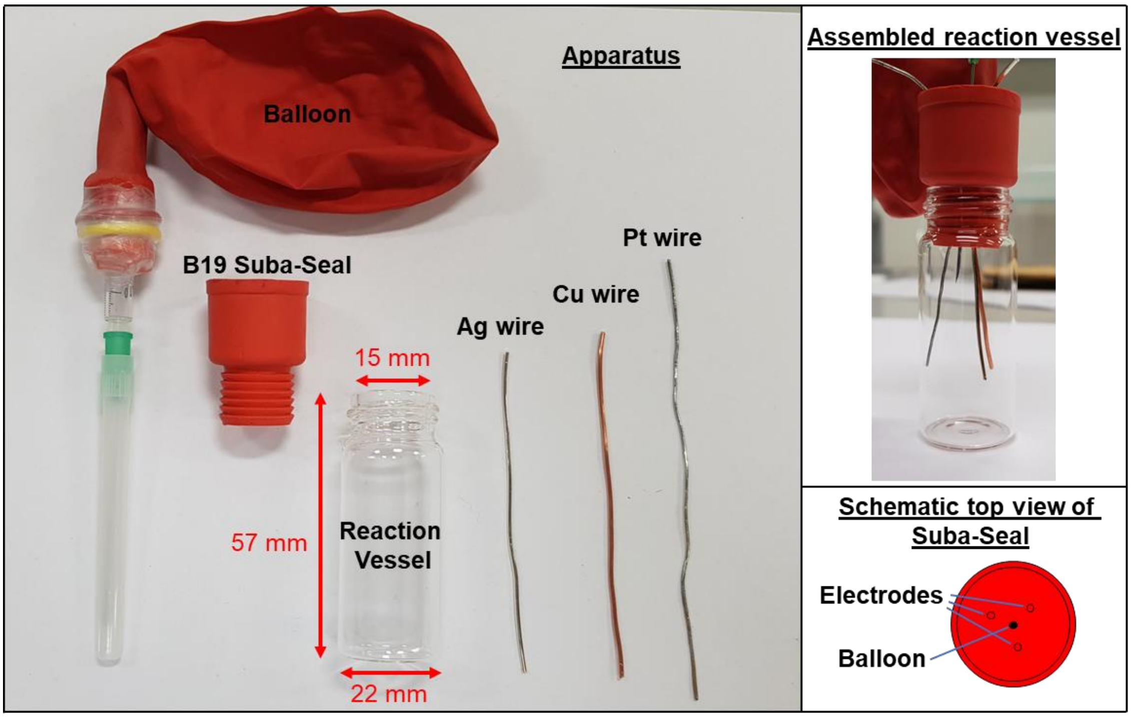

Figure S1. Image of electrochemical apparatus used with dimensions.

Electrochemical Preparation of Copper Acetylides:

Cyclic Voltammetry (CV) Plots of Phenylacetylene:

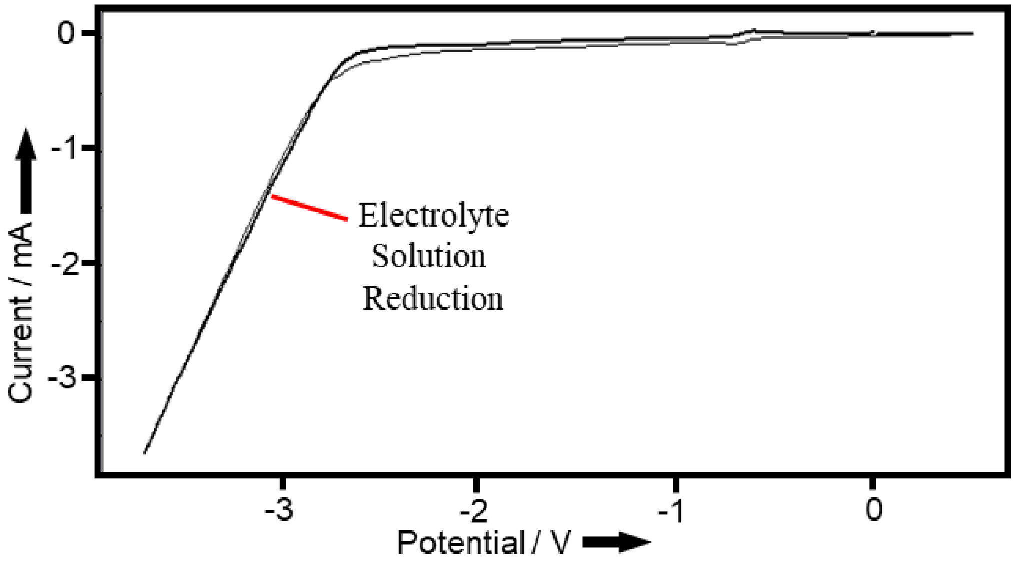

Figure S2. Background CV plot of 0.1 M $Et_4NO_3SC_6H_4CH_3$ / MeCN. Axes redrawn for clarity.

This plot indicates that without any other reagents present the background electrolyte solution will be reduced from around -3.0 V vs Ag QRE. This reduction appears irreversible in nature.

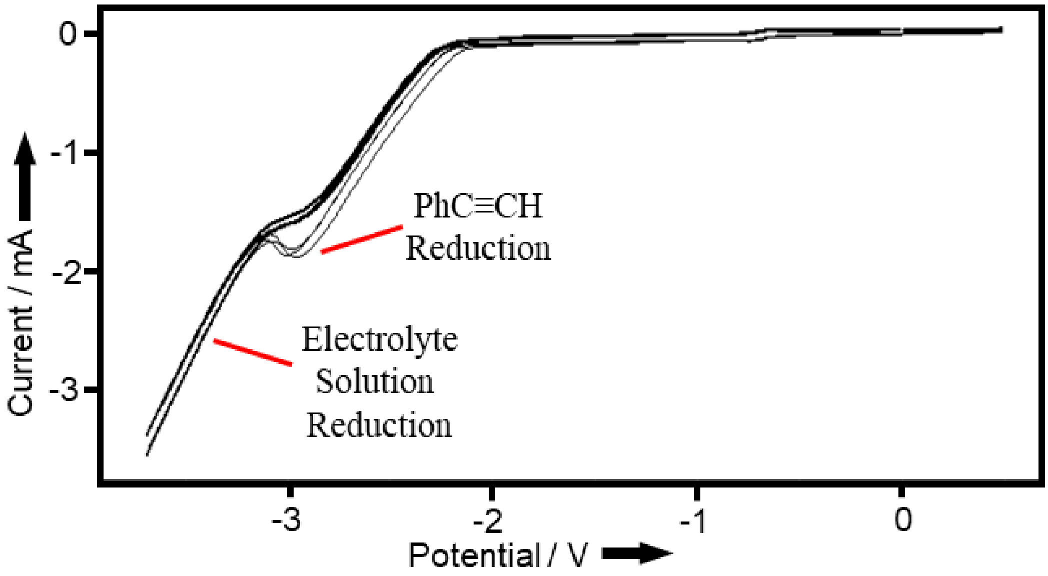

Figure S3. CV plot of phenylacetylene in 0.1 M $Et_4NO_3SC_6H_4CH_3$ / MeCN. Axes redrawn for clarity.

This plot indicates that when phenylacetylene is present it is reduced in favour of the background electrolyte solution (reduction peak from around -2.2 V vs Ag QRE compared to the -3.0 V seen in Figure S2). This reduction appears to be irreversible in nature.

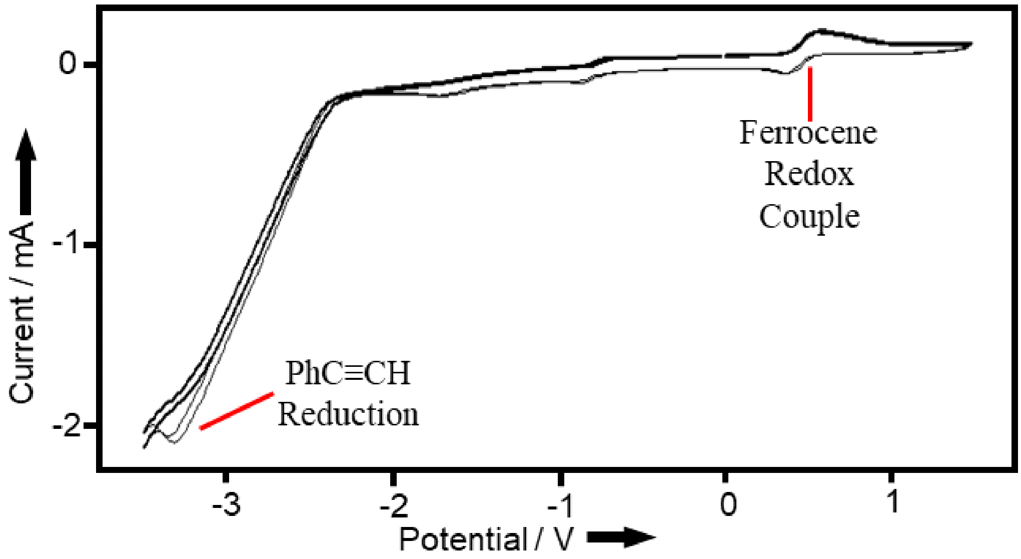

Figure S4. CV plot of phenylacetylene in 0.1 M $Et_4NO_3SC_6H_4CH_3$ / MeCN after ferrocene added for reference. Axes redrawn for clarity.

This plot shows the reductions seen in Figures S2 and S3 compared to the ferrocene redox couple. This acts as a convenient reference.

General Electrochemical Procedure for Copper(I) Acetylide Synthesis:

$Et_4N(O_3SC_6H_4CH_3)$ (0.03 g, 0.10 mmol, 1.0 equiv) was weighed out into a reaction vessel and sealed as shown in **Figure S1** with Cu wire (WE), Ag wire (QRE) and Pt wire (CE) electrodes pushed through the Suba-Seal. Anhydrous MeCN (10 mL) was then added to make up a 0.01 M solution. The terminal alkyne (0.20 mmol, 2.0 equiv) was then added *via* syringe and the solution was degassed with argon for 5 min. The electrodes were then connected up to a potentiostat and the voltage was set to run at +0.50 V for 4 h whilst stirring at RT under argon, immediately causing a yellow precipitate to form. The voltage was then stopped and the yellow precipitate was collected by Büchner filtration and washed with reagent grade MeCN (20 mL), then H_2O (20 mL), then acetone (20 mL) before being dried in a vacuum oven for 30 min to yield the dry copper acetylide product.

(Phenylethynyl)copper, 1a

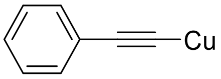

Phenylacetylene (32 mg, 0.32 mmol) used to yield a yellow solid (50 mg, 0.31 mmol, 97%); 45.7 C charge passed; m.p. 225-227 °C (dec.) (lit.,[1] 226-229 °C); IR ν_{max} (solid) 3045 (w), 2353 (w), 1929 (w), 1592 (w), 1569 (w), 1479 (m), 1439 (m), 743 (s), 681 (s), 520 (s), 510 (s) cm^{-1}. Data in agreement with literature.[1-4]

(*p*-Tolylethynyl)copper, 1b

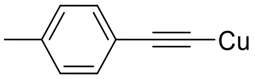

p-Tolylacetylene (25 mg, 0.21 mmol) used to yield a yellow solid (37 mg, 0.21 mmol, 96%); 31.2 C charge passed; m.p. 238-240 °C (dec.) (lit.,[4] 235-237 °C); IR ν_{max} (solid) 3016 (w), 2913 (m), 1930 (w), 1887 (w), 1500 (s), 805 (s), 516 (s) cm^{-1}. Data in agreement with literature.[2,4]

((4-(Trifluoromethyl)phenyl)ethynyl)copper, 1c

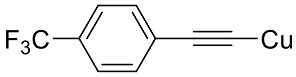

4-Ethynyl-α,α,α-trifluorophenylacetylene (31 mg, 0.18 mmol) used to yield a yellow solid (40 mg, 0.17 mmol, 95%); 27.1 C charge passed; m.p. 186-188 °C (dec.); IR ν_{max} (solid) 2920 (w), 1916 (m), 1607 (m), 1402 (m), 1315 (s), 1160 (m), 1113 (s), 1101 (s), 1063 (s), 1014 (s), 835 (s), 592 (m), 513 (m), 442 (m) cm^{-1}.

((2-(Trifluoromethyl)phenyl)ethynyl)copper, 1d

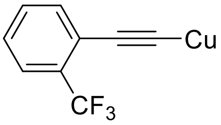

2-(Trifluoromethyl)-phenylacetylene (32 mg, 0.19 mmol) used to yield a bright yellow solid (43 mg, 0.18 mmol, 99%); 28.1 C charge passed; m.p. 243-245 °C (dec.); IR ν_{max} (solid) 3065 (w), 1930 (m), 1600 (m), 1570 (m), 1484 (m), 1445 (m), 1313 (s), 1157 (s), 1120 (s), 1107 (s), 1052 (s), 1031 (s), 756 (s), 744 (s), 650 (s), 530 (s) cm^{-1}.

(3-Ethoxy-3-oxoprop-1-yn-1-yl)copper, 1e

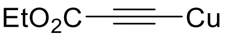

Ethyl propiolate (32 mg, 0.32 mmol) used to yield a bright yellow solid (26 mg, 0.16 mmol, 51%); 19.2 C charge passed; m.p. 181-183 °C (dec.) (lit.,[4] 182-184 °C); IR ν_{max} (solid) 2997 (w), 2928 (w), 1945 (s), 1927 (s), 1704 (s), 1662 (s), 1223 (s), 1020 (s), 747 (s) cm^{-1}. Data in agreement with literature.[4]

(4-Phenylbut-1-yn-1-yl)copper, 1f

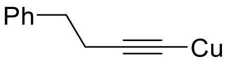

4-Phenyl-1-butyne (27 mg, 0.20 mmol) used to yield a yellow solid (25 mg, 0.13 mmol, 63%); 21.8 C charge passed; m.p. 174-175 °C (dec.) (lit.,[4] 174-176 °C); IR ν_{max} (solid) 3058 (w), 3025 (w), 2920 (w), 1599 (w), 1494 (m), 1452 (m), 744 (s), 696 (s) cm^{-1}. Data in agreement with literature.[4]

(Cyclohex-1-en-1-ylethynyl)copper, 1g

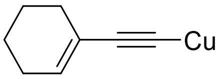

1-Ethynylcyclohexene (21 mg, 0.20 mmol) used to yield an orange solid (29 mg, 0.17 mmol, 86%); 23.9 C charge passed; m.p. 184-186 °C (dec.) (lit.,[4] 163-165 °C); IR ν_{max} (solid) 2923 (s), 2853 (s), 2825 (s), 1619 (w), 1432 (m), 916 (m), 838 (m), 798 (m), 615 (m), 532 (m) cm^{-1}. IR data firmly in agreement with literature but note that decomposition point varies significantly due to the large potential margin of error associated with the decomposition points of polymeric structures.[4]

((4-Methoxyphenyl)ethynyl)copper, 1h

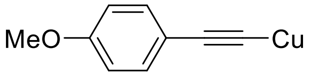

4-Ethynylanisole (25 mg, 0.19 mmol) used to yield a bright yellow solid (36 mg, 0.18 mmol, >99%); 19.3 C charge passed; m.p. 247-249 °C (dec.) (lit.,[5] 260 °C); IR ν_{max} (solid) 3031 (w), 2836 (w), 1600 (m), 1499 (s), 1247 (s), 1168 (s), 1029 (s), 818 (s), 533 (s), 449 (m) cm^{-1}. Data in agreement with literature.[3,5]

((Triisopropylsilyl)ethynyl)copper, 1i

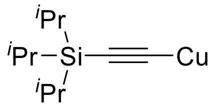

Triisopropylsilyl acetylene (37 mg, 0.20 mmol) used to yield a dark yellow solid (11 mg, 0.04 mmol, 21%); 14.4 C charge passed; m.p. 161-165 °C (dec.); IR ν_{max} (solid) 3315 (m), 2945 (w), 2867 (w), 1460 (m), 1372 (m), 883 (m), 835 (m), 613 (s), 451 (s) cm^{-1}.

(3,3-Dimethylbut-1-yn-1-yl)copper, 1j

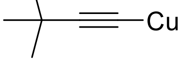

3,3-Dimethyl-1-butyne (16 mg, 0.19 mmol) used to yield a yellow solid (13 mg, 0.09 mmol, 47%); 16.6 C charge passed; m.p. 147-150 °C (dec.) (lit.,[6] 80-150 °C); IR ν_{max} (solid) 3322 (w), 2965 (m), 2923 (m),

2896 (m), 2864 (w), 2183 (w), 1471 (m), 1453 (m), 1360 (s), 1239 (s), 455 (s) cm^{-1}. Data in agreement with literature.[6]

<u>IR Spectra for Copper Acetylides:</u>

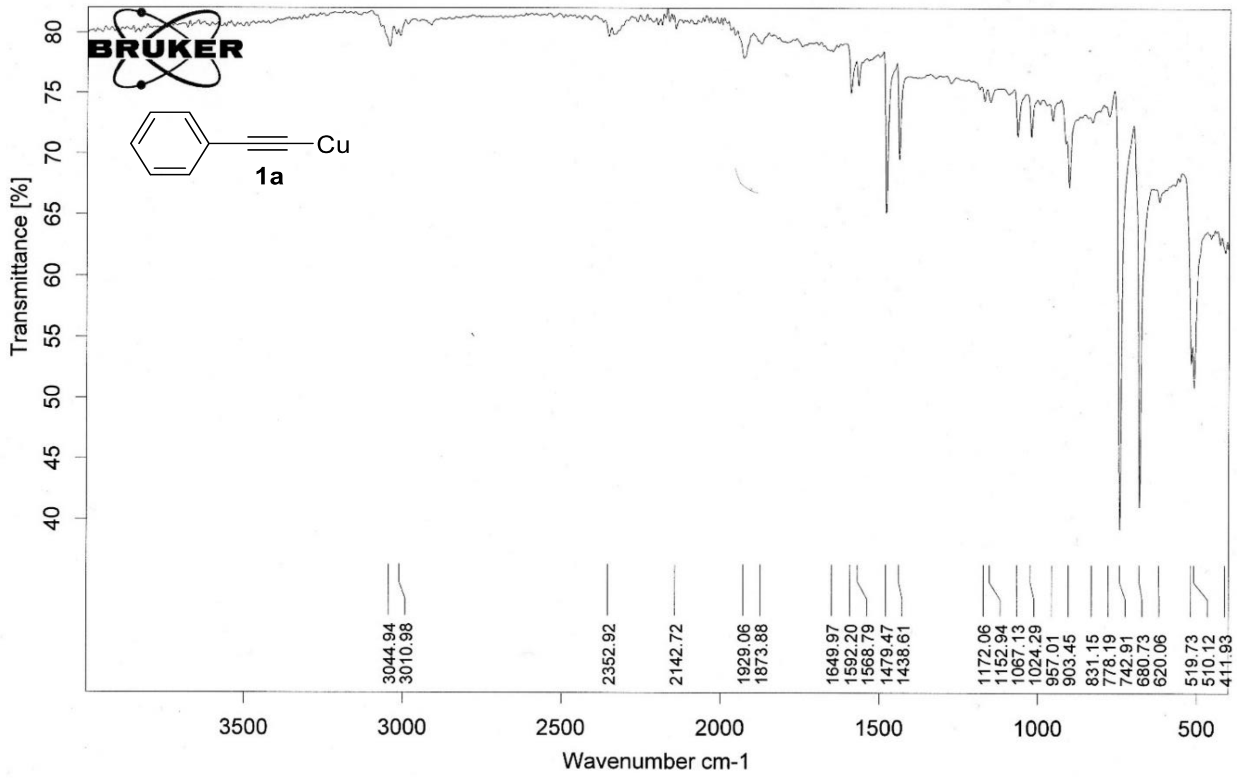

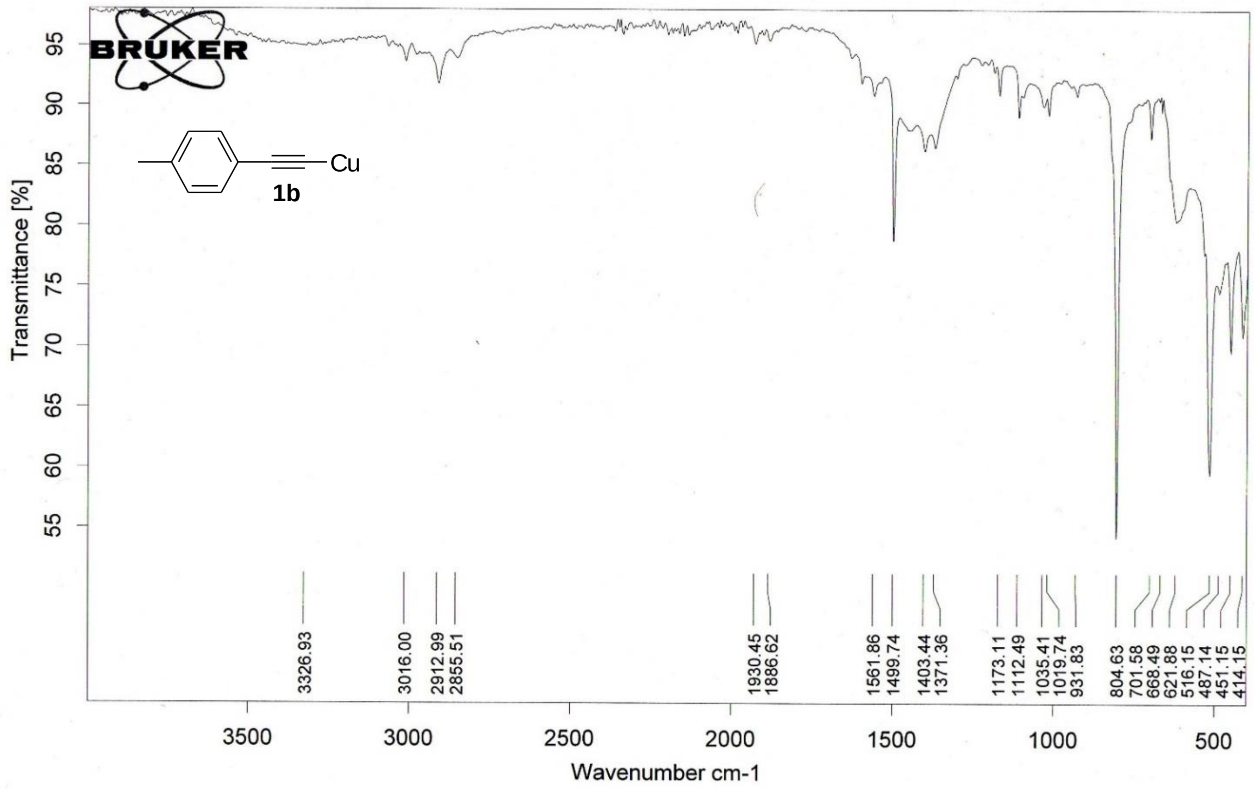

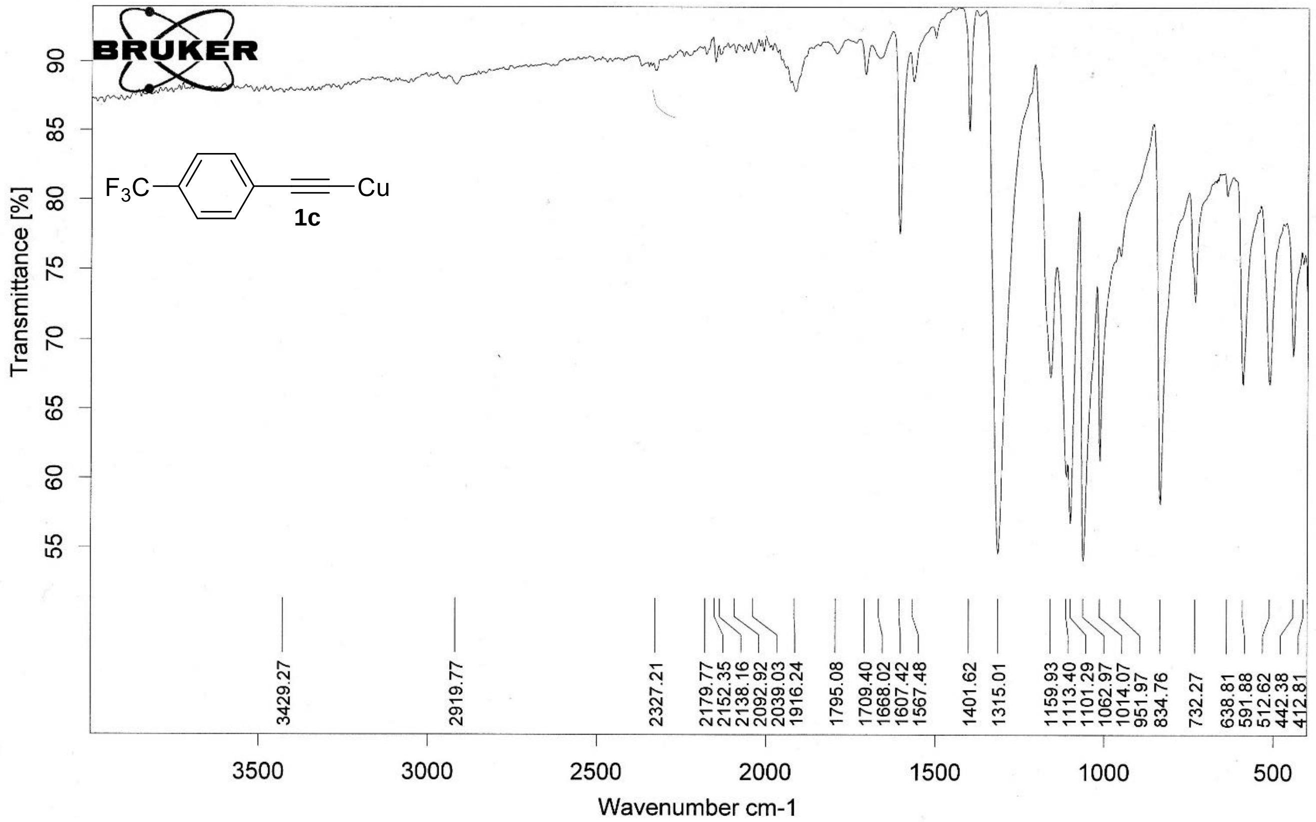
BRUKER
F3C
Cu
1c
Transmittance [%]
90
85
80
75
70
65
60
55
3429.27
2919.77
2327.21
2179.77
2152.35
2138.16
2092.92
2039.03
1916.24
1795.08
1709.40
1668.02
1607.42
1567.48
1401.62
1315.01
1159.93
1113.40
1101.29
1062.97
1014.07
951.97
834.76
732.27
638.81
591.88
512.62
442.38
412.81
3500
3000
2500
2000
1500
1000
500
Wavenumber cm-1

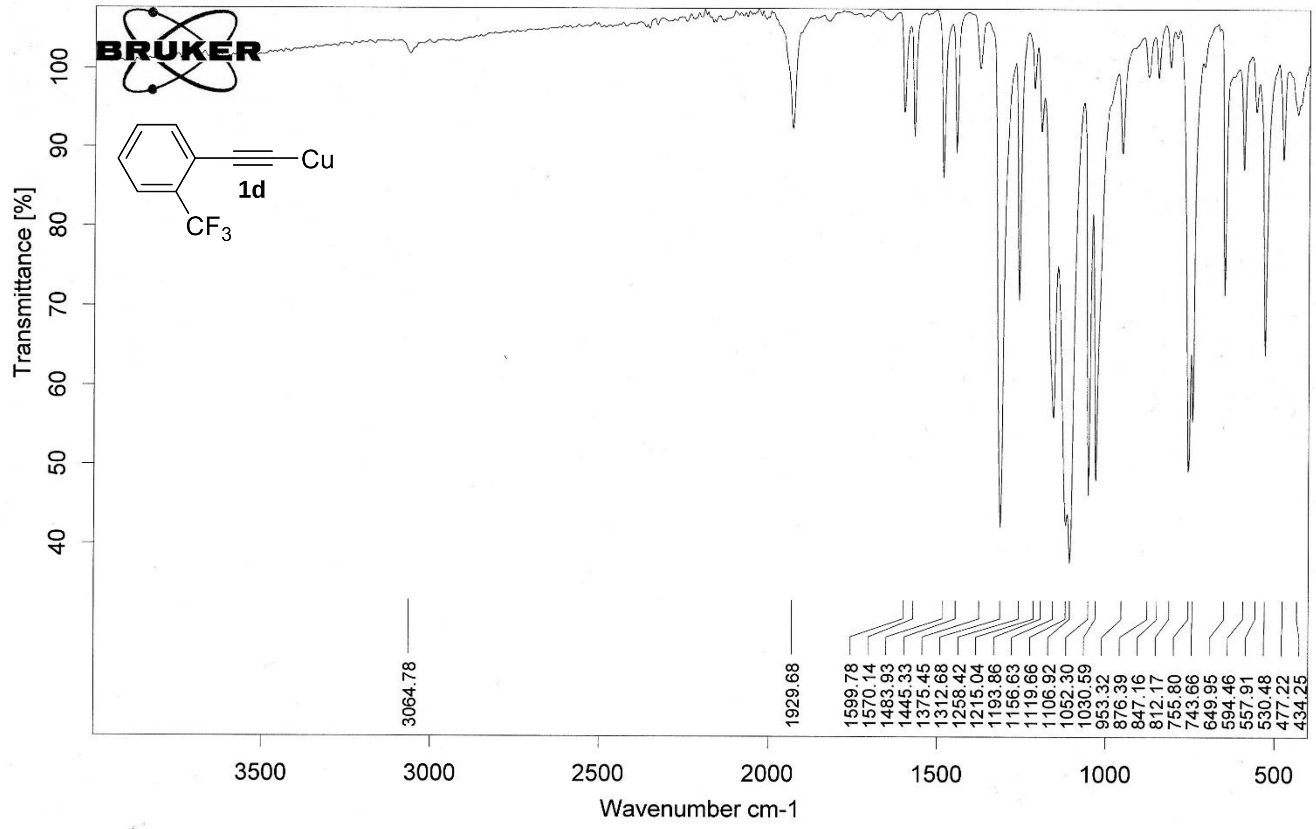
BRUKER
Cu
1d
CF3
Transmittance [%]
100
90
80
70
60
50
40
3064.78
1929.68
1599.78
1570.14
1483.93
1445.33
1375.45
1312.68
1258.42
1215.04
1193.86
1156.63
1119.66
1106.92
1052.30
1030.59
953.32
876.39
847.16
812.17
755.80
743.66
649.95
594.46
557.91
530.48
477.22
434.25
3500
3000
2500
2000
1500
1000
500
Wavenumber cm-1

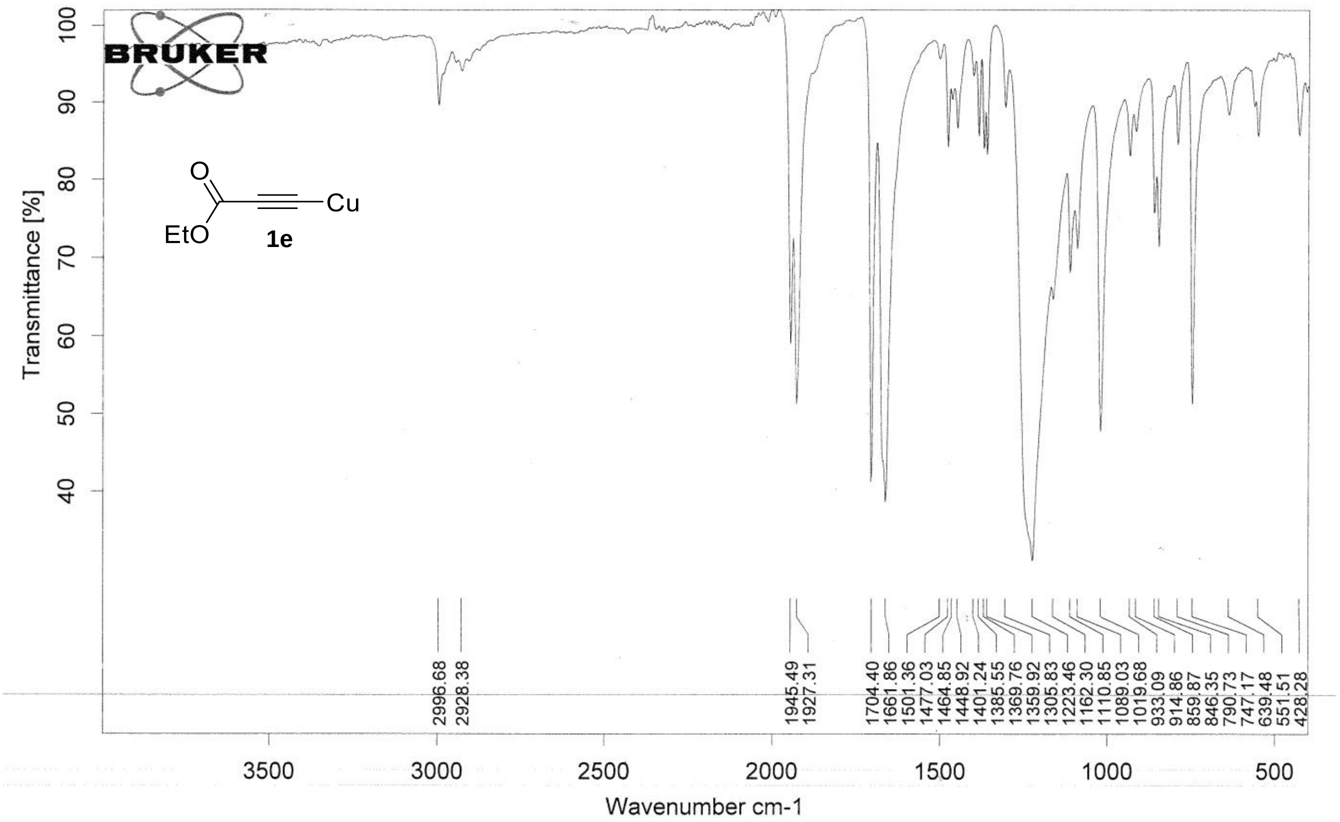
BRUKER
Transmittance [%]
Wavenumber cm-1
EtO
O
Cu
1e
2996.68
2928.38
1945.49
1927.31
1704.40
1661.86
1501.36
1477.03
1464.85
1448.92
1401.24
1385.55
1369.76
1359.92
1305.83
1223.46
1162.30
1110.85
1089.03
1019.68
933.09
914.86
859.87
846.35
790.73
747.17
639.48
551.51
428.28

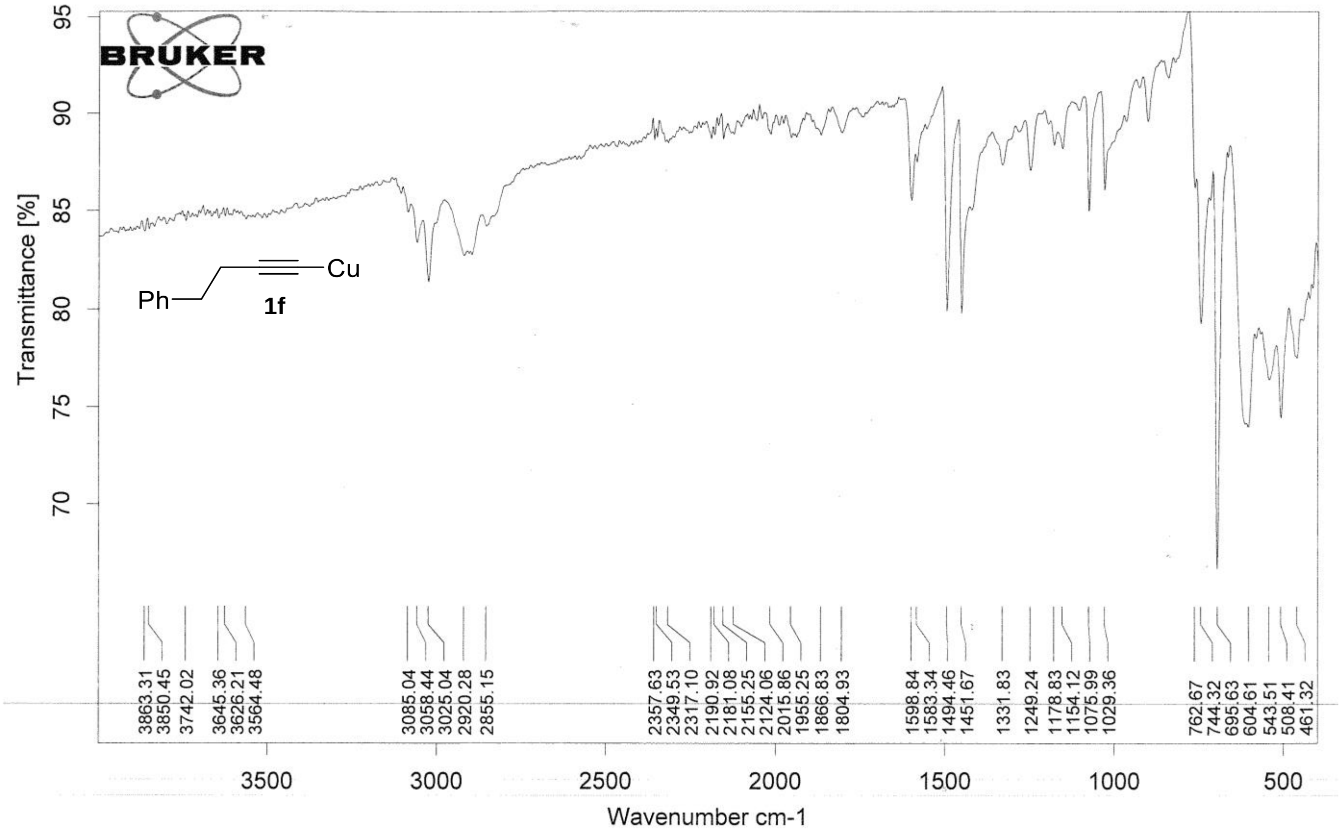
BRUKER
Transmittance [%]
Wavenumber cm-1
Ph
Cu
1f
3863.31
3850.45
3742.02
3645.36
3626.21
3564.48
3085.04
3058.44
3025.04
2920.28
2855.15
2357.63
2349.53
2317.10
2190.92
2181.08
2155.25
2124.06
2015.86
1955.25
1866.83
1804.93
1598.84
1583.34
1494.46
1451.67
1331.83
1249.24
1178.83
1154.12
1075.99
1029.36
762.67
744.32
695.63
604.61
543.51
508.41
461.32

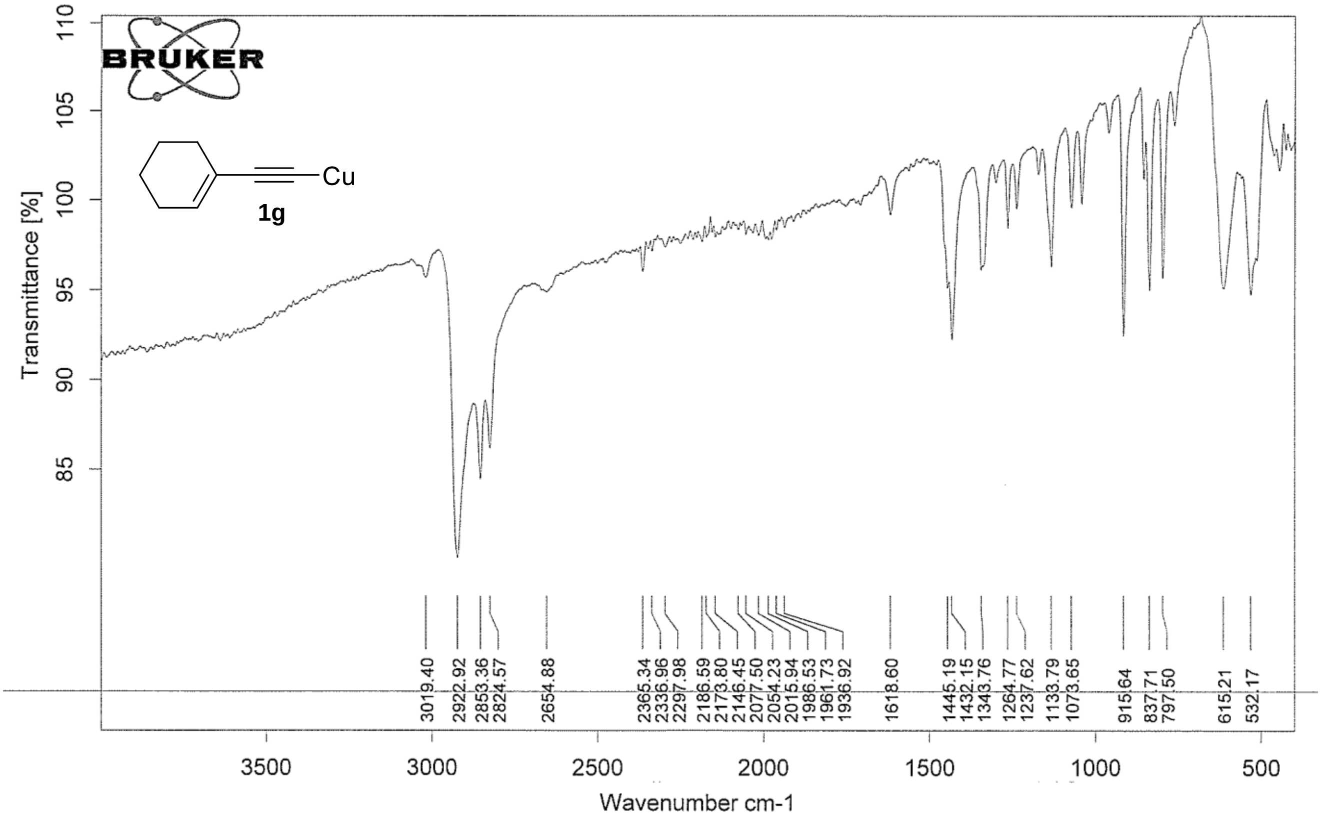
BRUKER
Cu
1g
Transmittance [%]
110
105
100
95
90
85
3019.40
2922.92
2853.36
2824.57
2654.88
2365.34
2336.96
2297.98
2186.59
2173.80
2146.45
2077.50
2054.23
2015.94
1986.53
1961.73
1936.92
1618.60
1445.19
1432.15
1343.76
1264.77
1237.62
1133.79
1073.65
915.64
837.71
797.50
615.21
532.17
3500
3000
2500
2000
1500
1000
500
Wavenumber cm-1

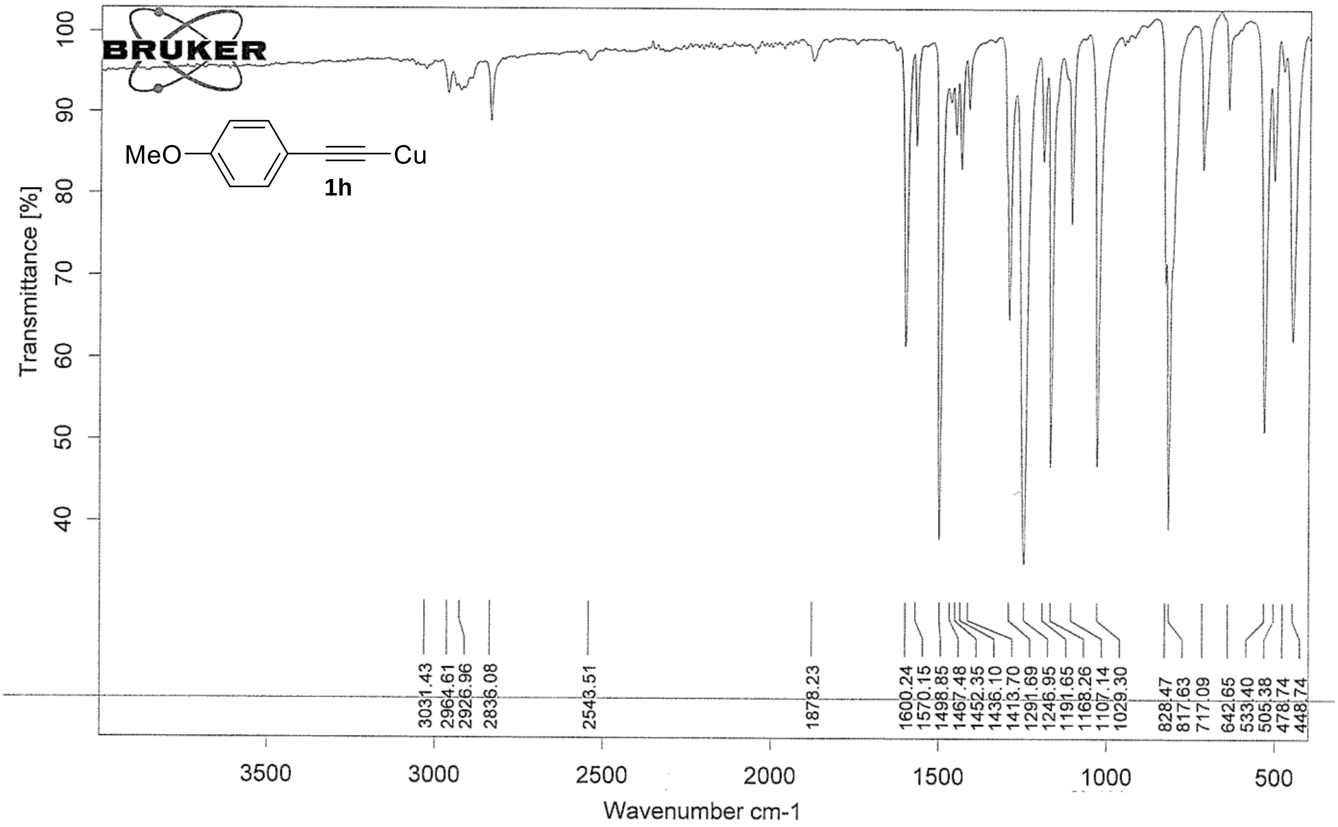
BRUKER
MeO
Cu
1h
Transmittance [%]
100
90
80
70
60
50
40
3031.43
2964.61
2926.96
2836.08
2543.51
1878.23
1600.24
1570.15
1498.85
1467.48
1452.35
1436.10
1413.70
1291.69
1246.95
1191.65
1168.26
1107.14
1029.30
828.47
817.63
717.09
642.65
533.40
505.38
478.74
448.74
3500
3000
2500
2000
1500
1000
500
Wavenumber cm-1

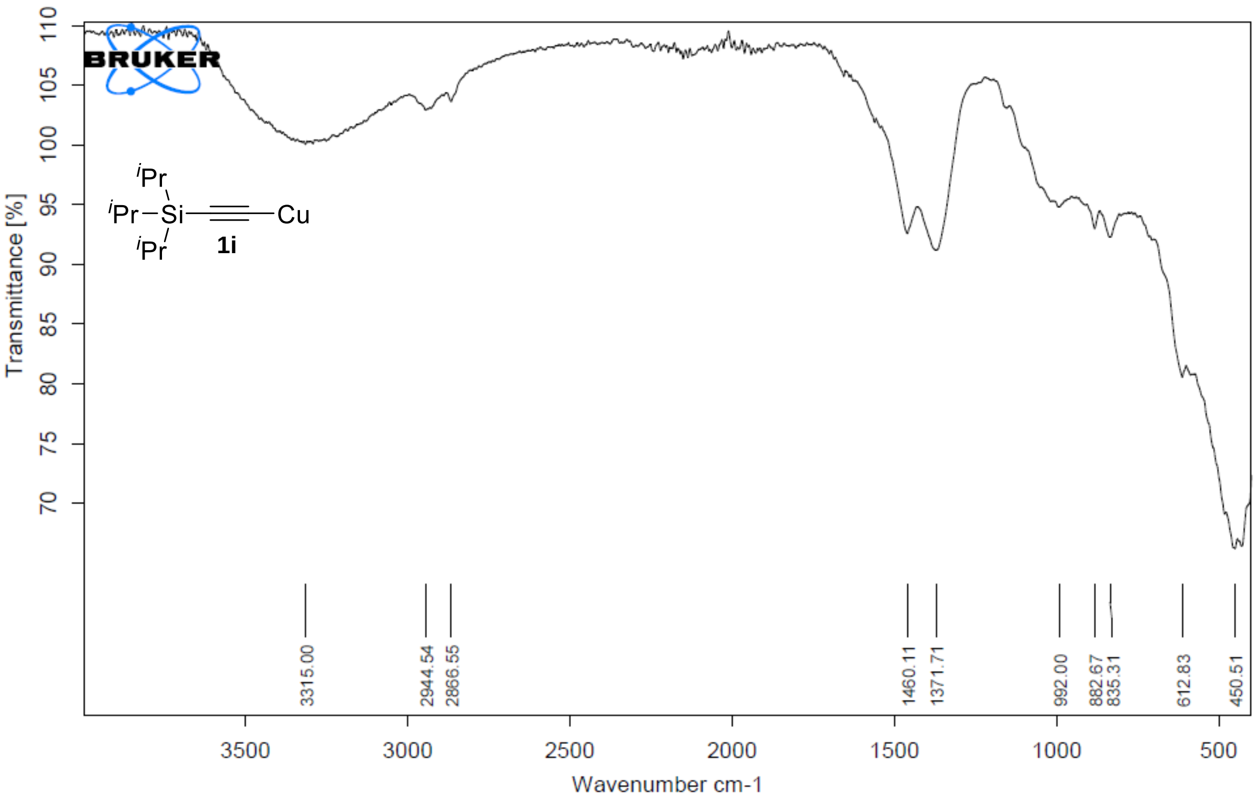
BRUKER
Transmittance [%]
110
105
100
95
90
85
80
75
70
^{i}Pr
^{i}Pr–Si≡Cu
^{i}Pr
1i
3315.00
2944.54
2866.55
1460.11
1371.71
992.00
882.67
835.31
612.83
450.51
3500
3000
2500
2000
1500
1000
500
Wavenumber cm-1

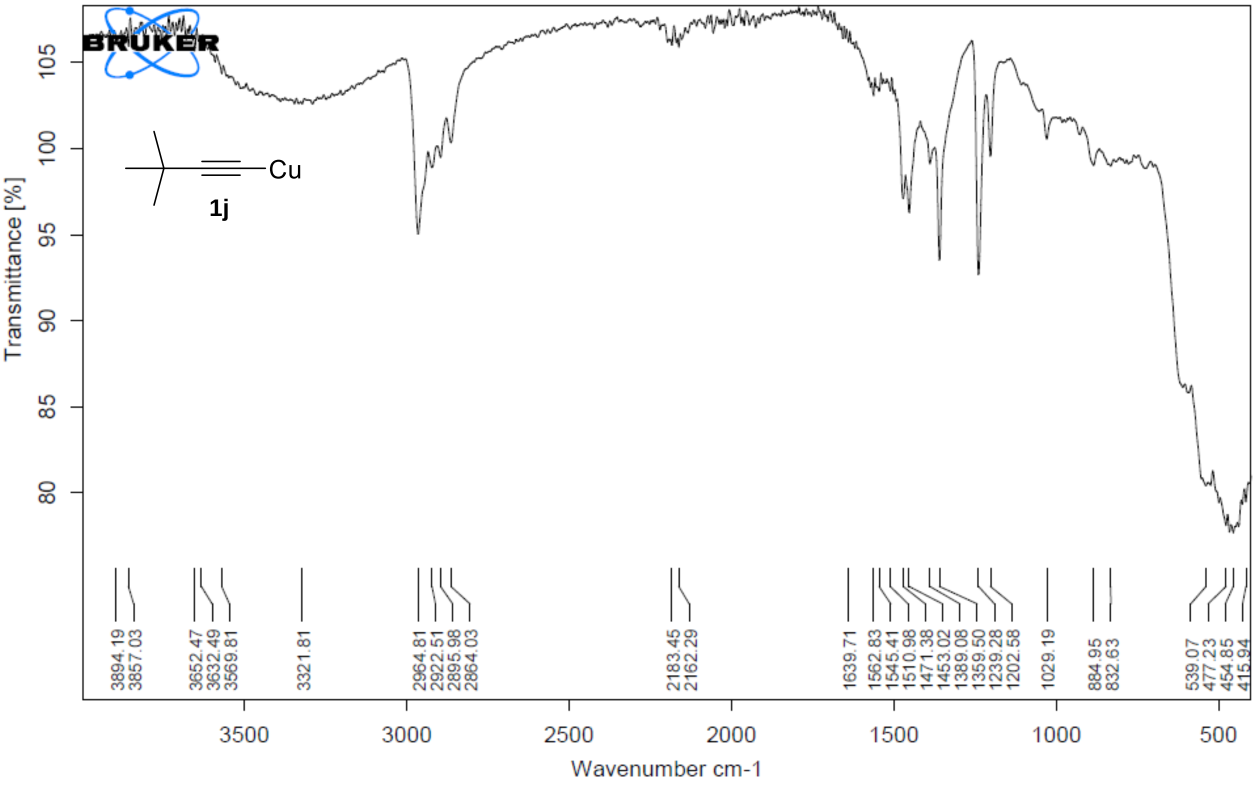
BRUKER
Transmittance [%]
105
100
95
90
85
80
Cu
1j
3894.19
3857.03
3652.47
3632.49
3569.81
3321.81
2964.81
2922.51
2895.98
2864.03
2183.45
2162.29
1639.71
1562.83
1545.41
1510.98
1471.38
1453.02
1389.08
1359.50
1239.28
1202.58
1029.19
884.95
832.63
539.07
477.23
454.85
415.94
3500
3000
2500
2000
1500
1000
500
Wavenumber cm-1

‘Click test’ of Copper(I) Acetylides Produced By Traditional Methods and Using the Electrochemical Method Developed in this Work:

Traditional Procedure for Copper(I) Acetylide **1a** Formation: (adapted from Evano *et al.*[7])

To a flask backfilled with argon, a mixture of ammonium hydroxide (30% solution, 50 mL), EtOH (30 mL) and CuI (3.80 g, 20 mmol, 2.0 equiv) was added to create a deep blue solution. Phenylacetylene (1.02 g, 10 mmol, 1.0 equiv) was then added dropwise whilst stirring to immediately cause a bright yellow preciptate to form. The solution was then left to stir at RT under argon for 16 h, before the precipitate was collected by Büchner filtration and washed successively with ammonium hydroxide (10% solution, 100 mL), then H_2O (50 mL), then EtOH (30 mL) and finally Et_2O (100 mL). The bright yellow solid was then dried in a vacuum oven for 2 h to yield the dry product **1a** as a bright yellow solid (1.64 g, 10 mmol, 99%).

General Procedure for 1,2,3-Triazole Formation: (adapted from Shao *et al.*[8])

A flask was charged with copper(I) acetylide **1a** (1.0 equiv), followed by BnN_3 (1.5 equiv) dissolved in reagent grade cyclohexane (3 mL). The solution was stirred at RT as glacial acetic acid (1.0 equiv) was added dropwise, causing an immediate colour change from yellow to pale green. The flask was then sealed with a Suba-Seal and degassed for 3 min, before being left to stir at RT under argon for 16 h. The solution was then diluted with EtOAc (15 mL) and filtered into a separating funnel, before being washed with 1 M $HCl_{(aq.)}$ (10 mL), then 1 M $K_2CO_{3(aq.)}$ (10 mL), then H_2O (15 mL). The organic layer was then dried over Na_2SO_4, filtered and concentrated *in vacuo*. The crude off-white solid was then purified by column chromatography (25% EtOAc/pet. ether.) to give the pure product **5** as a white crystalline solid.

1-Benzyl-4-phenyl-1*H*-1,2,3-triazole, 5

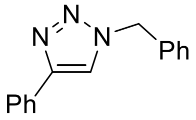

A. Copper(I) acetylide **1a** from traditional method (0.11 g, 0.67 mmol) used to yield **5** as a white crystalline solid (0.12 g, 0.50 mmol, 75%); m.p. 128-129 °C (lit.,[8] 130-131 °C); R_f 0.20 (25% EtOAc/pet. ether.); IR ν_{max} (solid) 3121 (w), 3095 (w), 3063 (w), 3033 (w), 2923 (w), 1466 (m), 1205 (m), 1028 (m), 764 (s), 726 (s), 691 (s) cm^{-1}; ^{1}H NMR (600 MHz, $CDCl_3$) δ_H 7.79-7.81 (2H, m, Ar*H*), 7.65 (1H, s, N-C*H*), 7.35-7.41 (5H, m, Ar*H*), 7.30-7.33 (3H, m, Ar*H*), 5.58 (2H, s, N-CH_2) ppm; ^{13}C NMR (100 MHz, $CDCl_3$) δ_C 148.4 (*C*), 134.8 (*C*), 130.7 (*C*), 129.2 (*C*H), 128.9 (*C*H), 128.2 (*C*H), 128.1 (*C*H), 125.8 (*C*H), 119.5 (*C*H), 54.3 (*C*H_2) ppm; LRMS (ESI) m/z 236 ($[M+H]^+$, 100%), 143 (7%), 91 (7%), 83 (10%). Data in agreement with literature.[8,9]

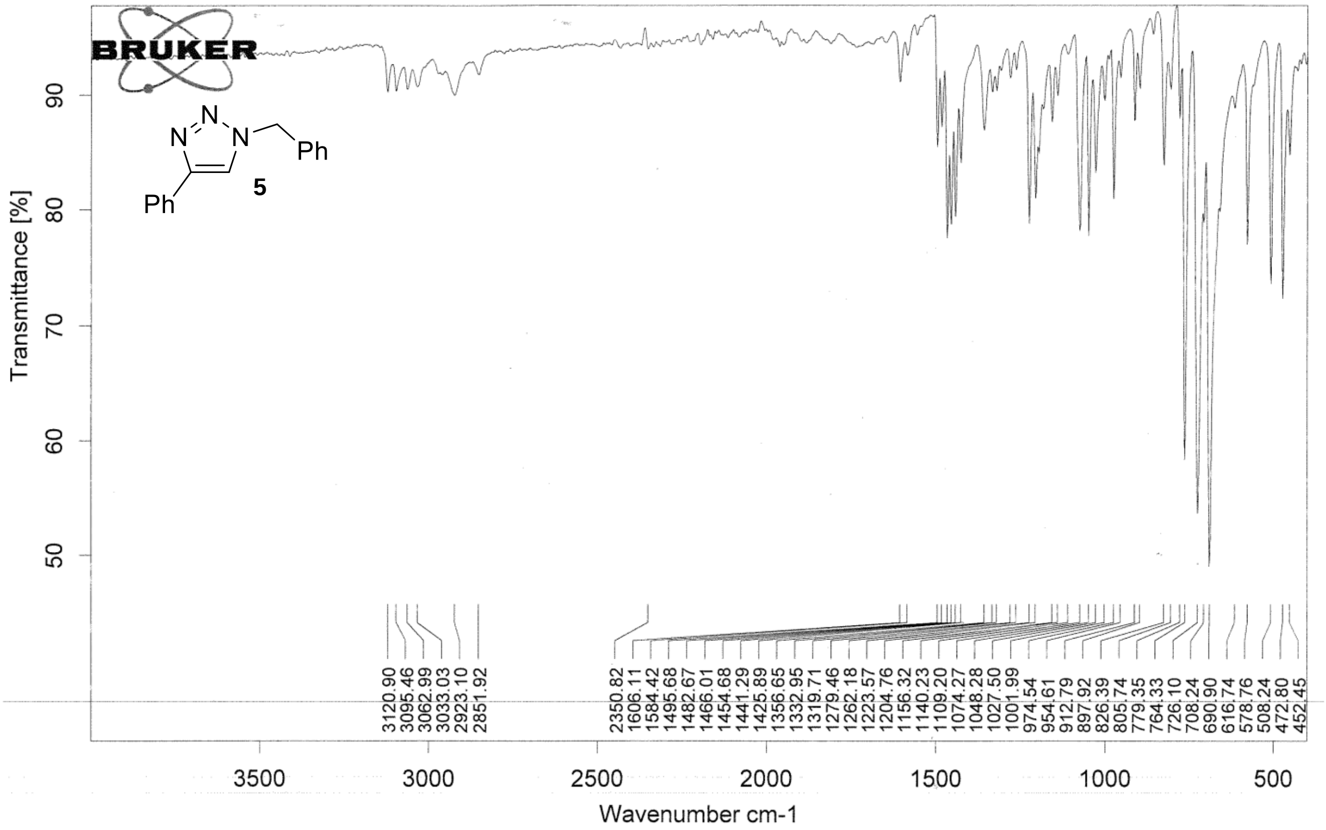
BRUKER
Ph
N
N
N
Ph
5
Transmittance [%]
90
80
70
60
50
3120.90
3095.46
3062.99
3033.03
2923.10
2851.92
2350.82
1606.11
1584.42
1495.68
1482.67
1466.01
1454.68
1441.29
1425.89
1356.65
1332.95
1319.71
1279.46
1262.18
1223.57
1204.76
1156.32
1140.23
1109.20
1074.27
1048.28
1027.50
1001.99
974.54
954.61
912.79
897.92
826.39
805.74
779.35
764.33
726.10
708.24
690.90
616.74
578.76
508.24
472.80
452.45
3500
3000
2500
2000
1500
1000
500
Wavenumber cm-1

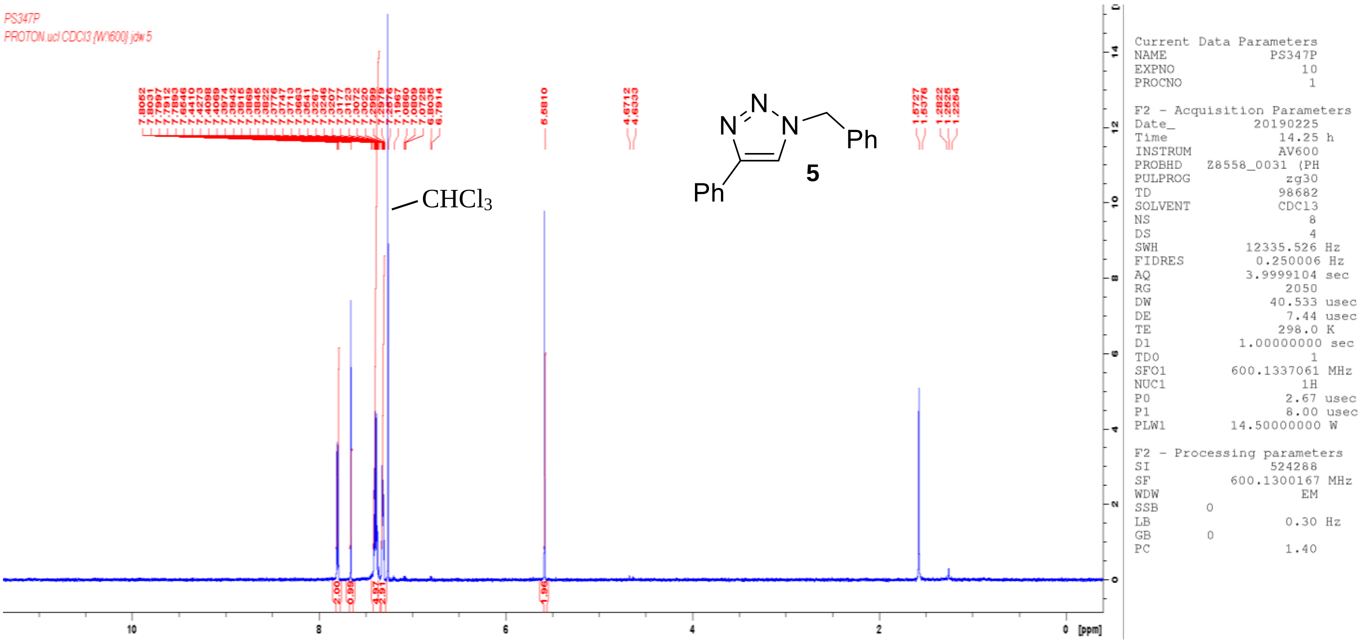
PS347P
PROTON.ucl CDCl3 {W:\600} jdw 5
CHCl3
Ph
N
N
N
Ph
5
Current Data Parameters
NAME PS347P
EXPNO 10
PROCNO 1
F2 - Acquisition Parameters
Date_ 20190225
Time 14.25 h
INSTRUM AV600
PROBHD Z8558_0031 (PH
PULPROG zg30
TD 98682
SOLVENT CDCl3
NS 8
DS 4
SWH 12335.526 Hz
FIDRES 0.250006 Hz
AQ 3.9999104 sec
RG 2050
DW 40.533 usec
DE 7.44 usec
TE 298.0 K
D1 1.00000000 sec
TD0 1
SFO1 600.1337061 MHz
NUC1 1H
P0 2.67 usec
P1 8.00 usec
PLW1 14.50000000 W
F2 - Processing parameters
SI 524288
SF 600.1300167 MHz
WDW EM
SSB 0
LB 0.30 Hz
GB 0
PC 1.40
10
8
6
4
2
0
[ppm]

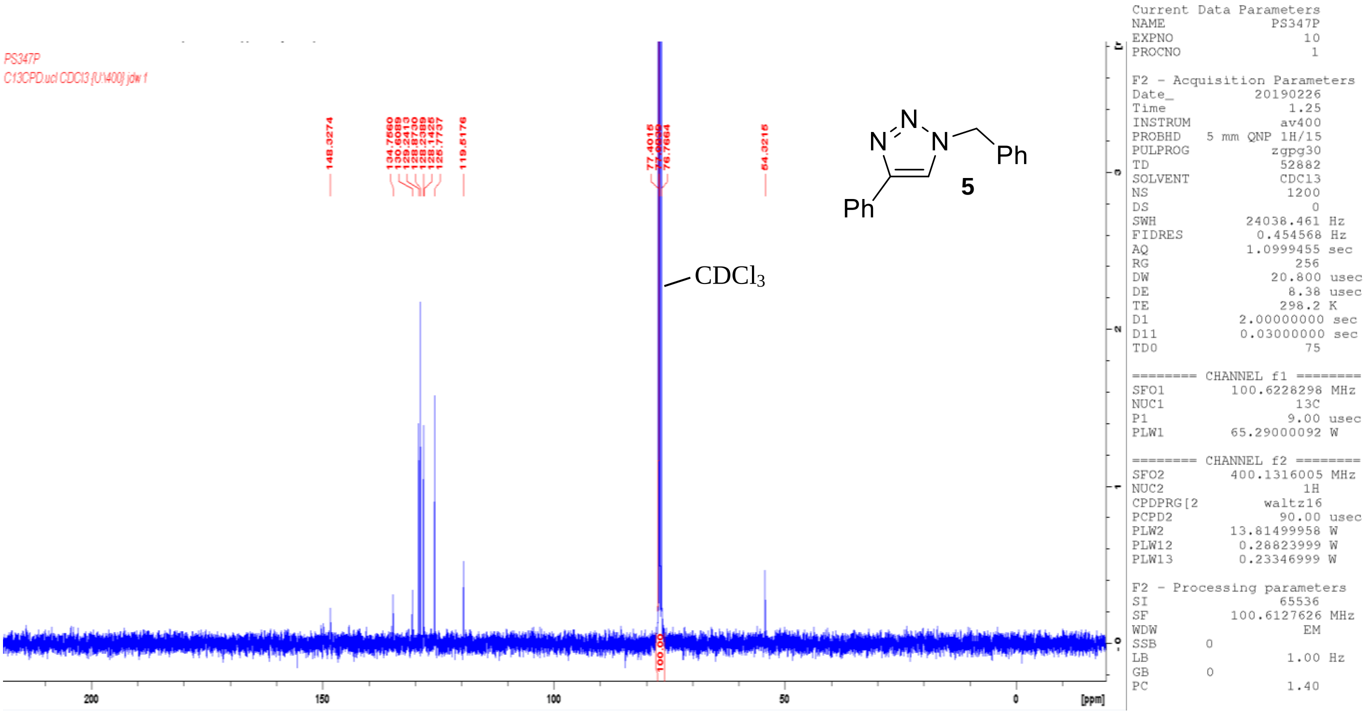

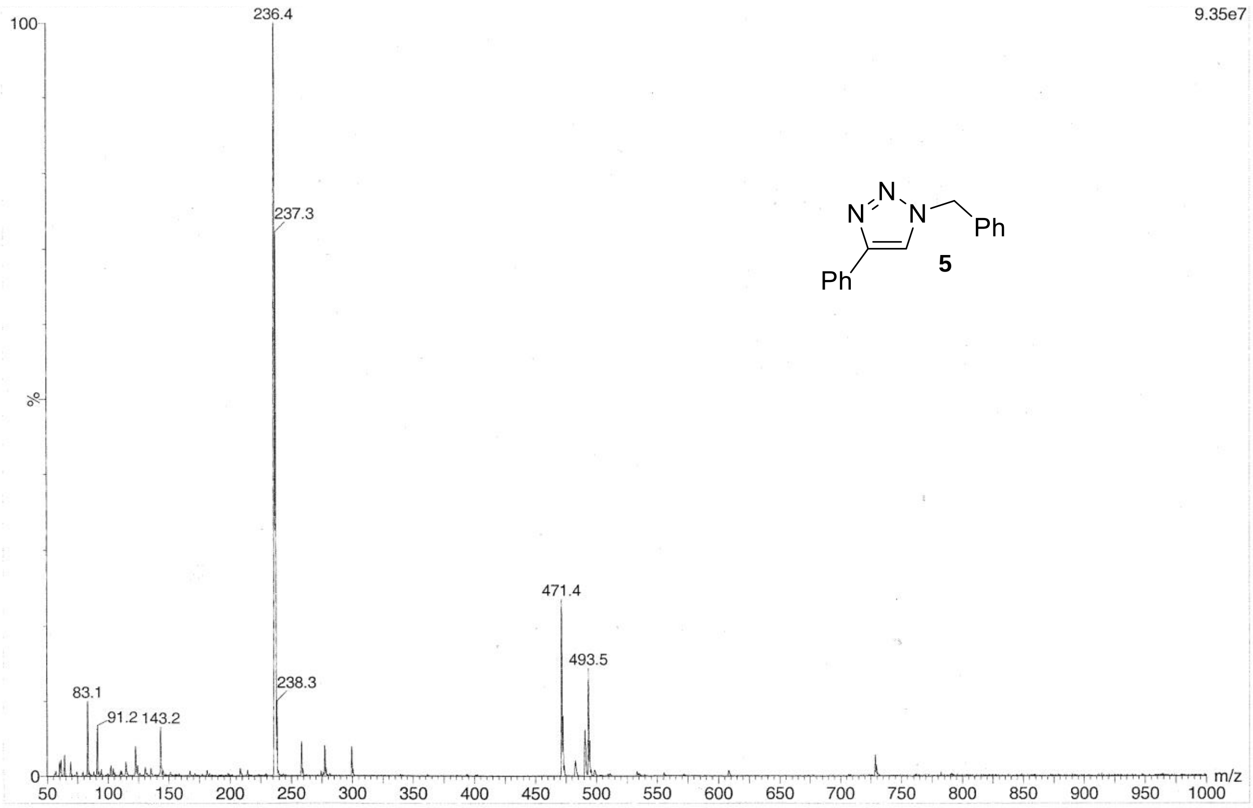

B. Copper(I) acetylide **1a** from electrochemical method developed in this work (58 mg, 0.35 mmol) used to yield **5** as a white crystalline solid (60 mg, 0.25 mmol, 72%); m.p. 127-128 °C (lit.,[8] 130-131 °C); R_f 0.20 (25% EtOAc/pet. ether.); IR ν_{max} (solid) 3142 (w), 2976 (w), 2924 (w), 2853 (w), 1450 (m), 1223 (m), 1045 (m), 767 (s), 727 (s), 694 (s) cm^{-1}; ^{1}H NMR (600 MHz, $CDCl_3$) δ_H 7.79-7.81 (2H, m, Ar*H*), 7.65 (1H, s, N-C*H*), 7.35-7.41 (5H, m, Ar*H*), 7.30-7.33 (3H, m, Ar*H*), 5.58 (2H, s, N-C*H*$_2$) ppm; ^{13}C NMR (100 MHz, $CDCl_3$) δ_C 148.3 (*C*), 134.8 (*C*), 130.6 (*C*), 129.2 (*C*H), 128.9 (*C*H), 128.2 (*C*H), 128.1

(*C*H), 125.8 (*C*H), 119.5 (*C*H), 54.4 (*C*H_2) ppm; LRMS (ESI) m/z 236 ($[M+H]^+$, 100%). Data in agreement with literature.[8,9]

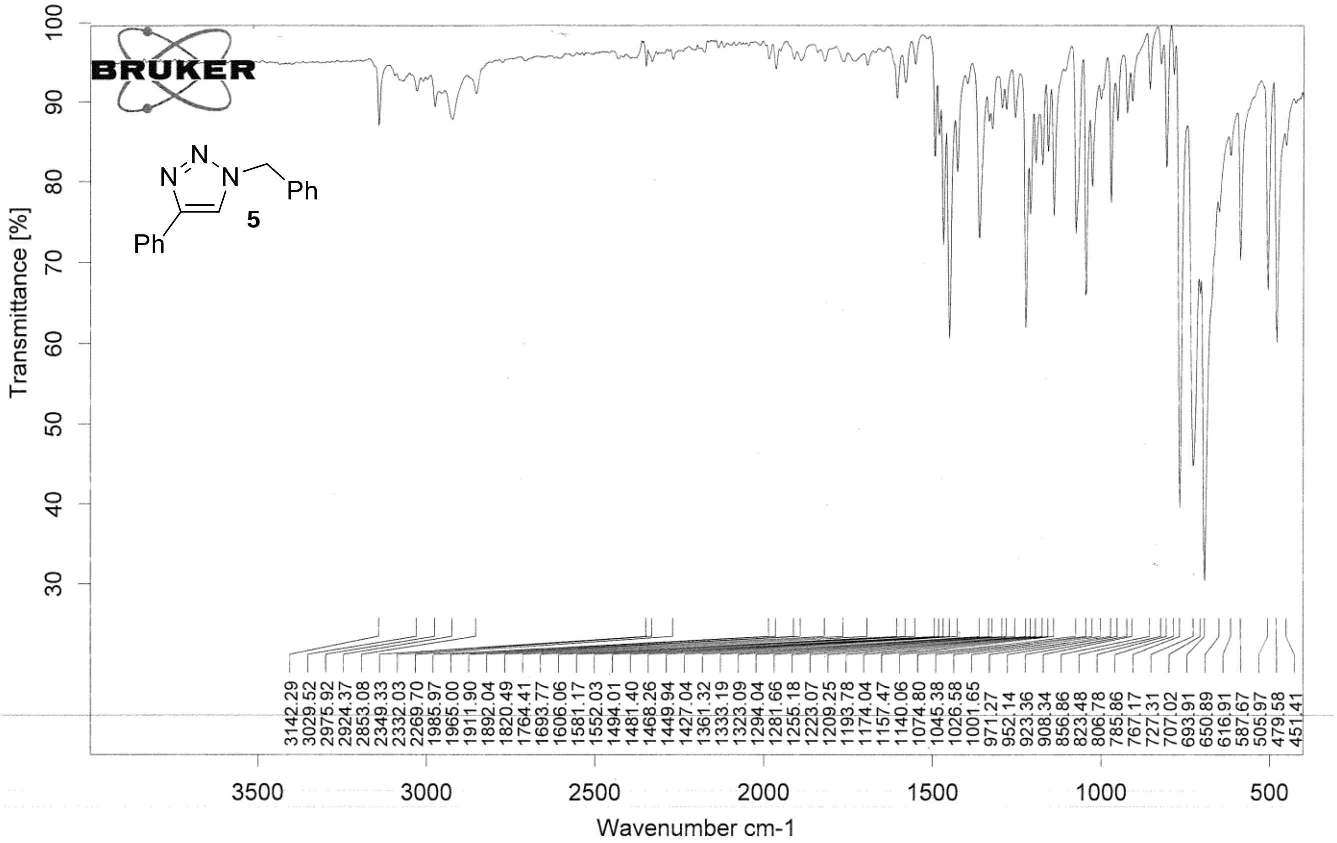

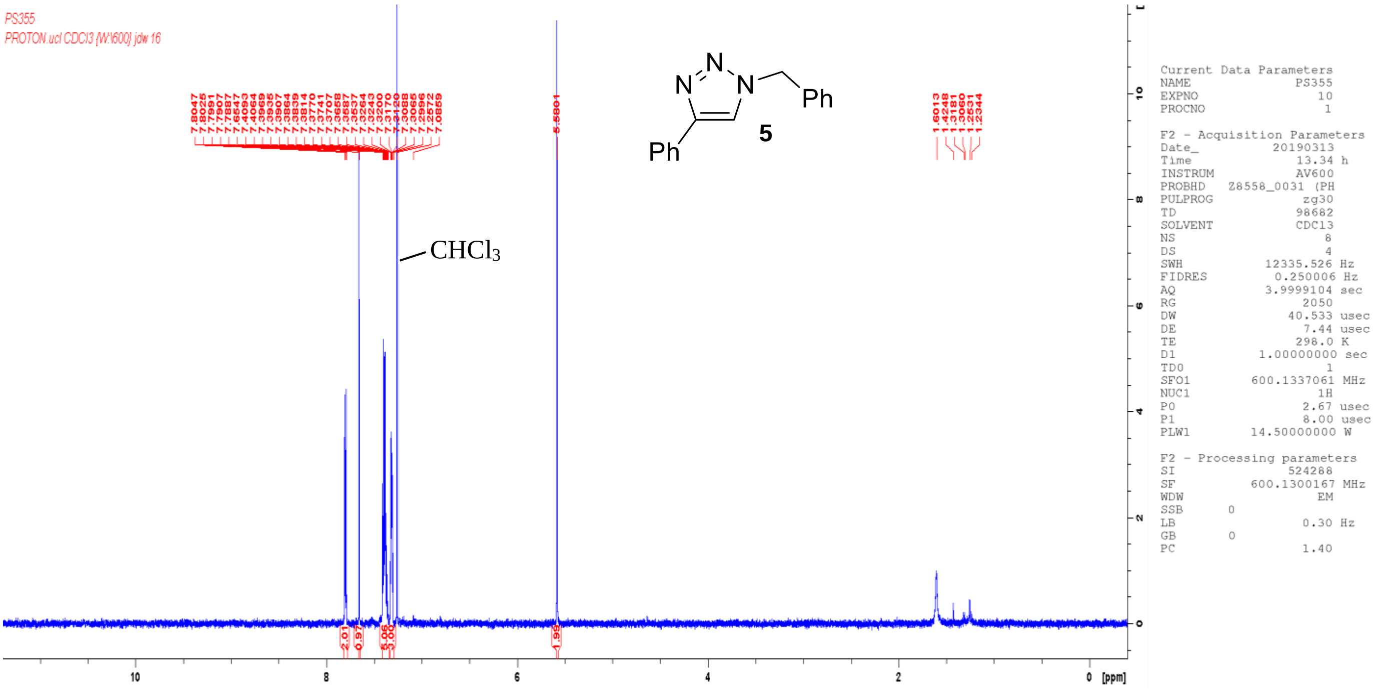

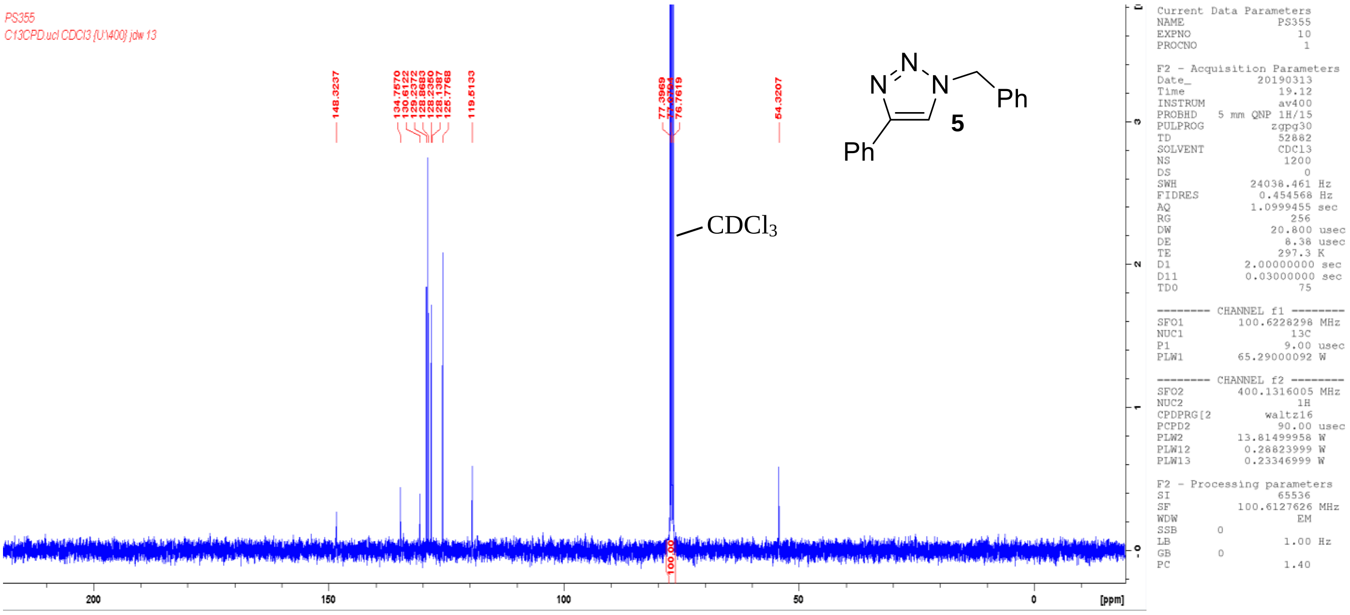

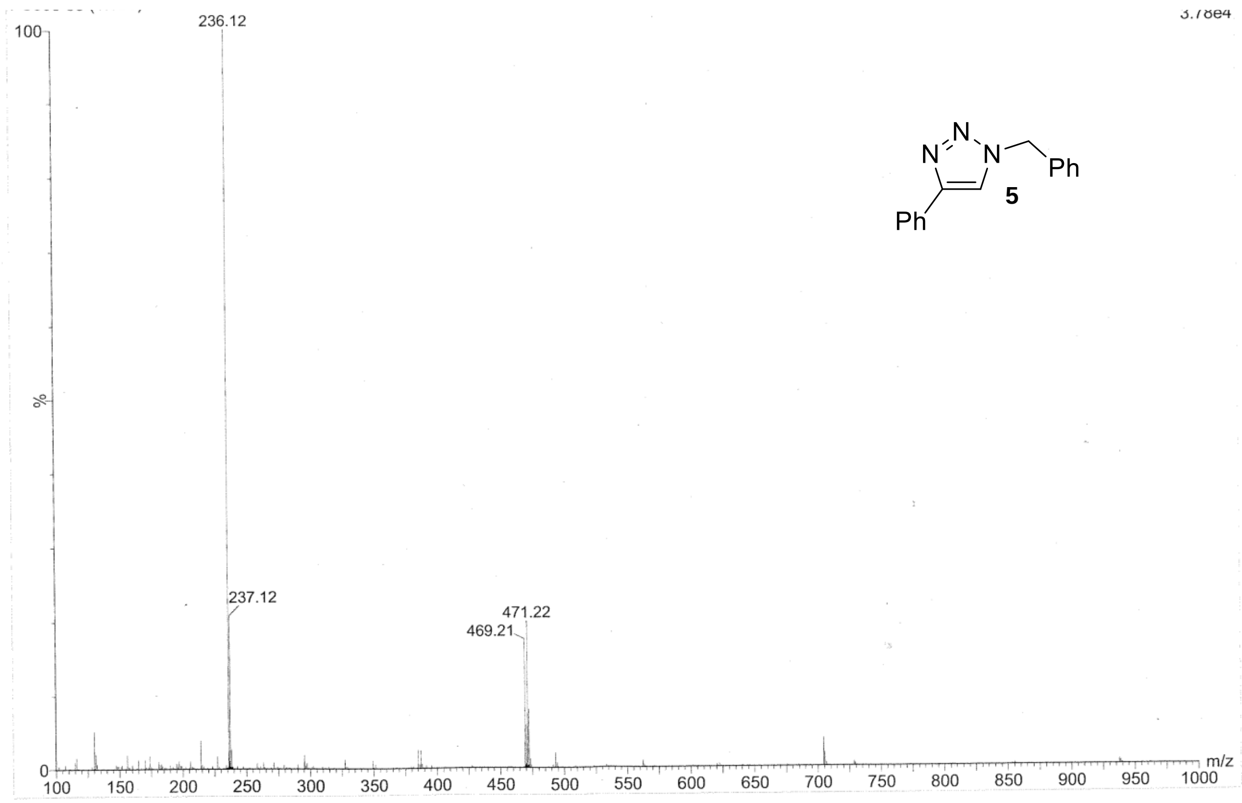

Good agreement between the two data sets.

Electrochemical Procedure for One-Pot Click Reaction:

Electrolyte salt (0.10 mmol, 0.5 equiv) was weighed out into a reaction vessel and sealed as shown in **Figure S1** with Cu wire (WE), Ag wire (QRE) and Pt wire (CE) electrodes pushed through the Suba-Seal. Anhydrous MeCN (10 mL) was then added to make up a 0.01 M solution. Phenylacetylene (0.20 mmol, 1.0 equiv) and BnN_3 (0.30 mmol, 1.5 equiv) were then added *via* syringe and the solution was degassed with argon for 5 min. The electrodes were then connected up to a potentiostat and the voltage was set to run at +0.50 V for 3 h whilst stirring at RT under argon. The voltage was then stopped and the solution was left to stir for a further 13 h at RT, under argon, before being diluted with EtOAc (15 mL) and filtered into a separating funnel. The organic layer was then washed with 1 M $HCl_{(aq.)}$ (10 mL), then 1 M $K_2CO_{3(aq.)}$ (10 mL) and dried over Na_2SO_4 before being filtered and concentrated *in vacuo*. The crude off-white solid was then purified by column chromatography (25% EtOAc/pet. ether.) to yield the pure product **5** as a white crystalline solid.

$Et_4NO_3SC_6H_4CH_3$ (31 mg), phenylacetylene (22 mg, 0.22 mmol), BnN_3 (40 mg), 28.42 C charge passed, which equates to 136.76 mol% Cu generated, gave **5** (25 mg, 0.11 mmol, 49%).
$Et_4NOAc\cdot 4H_2O$ (27 mg), phenylacetylene (22 mg, 0.22 mmol), BnN_3 (43 mg), 7.146 C charge passed, which equates to 33.77 mol% Cu generated, gave **5** (41 mg, 0.17 mmol, 79%).

Control reactions using Cu(I)OAc:

Cu(I)OAc (26 mg) was weighed out into a reaction vessel, then anhydrous MeCN (10 mL) was added, followed by Et_3N (11 mg, 0.11 mmol), then BnN_3 (40 mg), then phenylacetylene (21 mg, 0.21 mmol). The solution was then degassed and left to stir at RT under argon for 16 h, before being diluted with EtOAc (15 mL) and filtered into a separating funnel. The organic layer was then washed with 1 M $HCl_{(aq.)}$ (10 mL), then 1 M $K_2CO_{3(aq.)}$ (10 mL) and dried over Na_2SO_4 before being filtered and concentrated *in vacuo*. The crude off-white solid was then purified by column chromatography (25% EtOAc/pet. ether.) to yield the pure product **5** (28 mg, 0.12 mmol, 57%) as a white crystalline solid.

Cu(I)OAc (26 mg) was weighed out into a reaction vessel, then anhydrous MeCN (10 mL) was added, followed by Et_3N (22 mg, 0.22 mmol), then BnN_3 (40 mg), then phenylacetylene (23 mg, 0.23 mmol). The solution was then degassed and left to stir at RT under argon for 16 h, before being diluted with EtOAc (15 mL) and filtered into a separating funnel. The organic layer was then washed with 1 M $HCl_{(aq.)}$ (10 mL), then 1 M $K_2CO_{3(aq.)}$ (10 mL) and dried over Na_2SO_4 before being filtered and concentrated *in vacuo*. The crude off-white solid was then purified by column chromatography (25% EtOAc/pet. ether.) to yield the pure product **5** (31 mg, 0.13 mmol, 59%) as a white crystalline solid.

References:

1 J. Comas-Barcelo, R. S. Foster, B. Fiser, E. Gomez-Bengoa and J. P. A. Harrity, *Chem. Eur. J.*, 2015, **21**, 3257-3263.
2 C. Theunissen, M. Lecomte, K. Jouvin, A. Laoiti, C. Guissart, J. Heimburger, E. Loire and G. Evano, *Synthesis*, 2014, **46**, 1157-1166.
3 D. Gallego, A. Brück, E. Irran, F. Meier, M. Kaupp, M. Driess and J. F. Hartwig, *J. Am. Chem. Soc.*, 2013, **135**, 15617-15626.
4 P. W. Seavill, K. B. Holt and J. D. Wilden, *Green Chem.*, 2018, **20**, 5474-5478.
5 Y. Okamoto and S. K. Kundu, *J. Phys. Chem.*, 1973, **77**, 2677-2680.
6 H. O. House and M. J. Umen, *J. Org. Chem.*, 1973, **38**, 3893-3901.
7 K. Jouvin, J. Heimburger and G. Evano, *Chem. Sci.*, 2012, **3**, 756-760.
8 C. Shao, G. Cheng, D. Su, J. Xu, X. Wang and Y. Hu, *Adv. Synth. Catal.*, 2010, **352**, 1587-1592.
9 J. M. Perez, R. Cano and D. J. Ramon, *RSC Adv.*, 2014, **4**, 23943-23951.
